# Serum- and Glucocorticoid-Inducible Kinase-1 (SGK-1) Plays a Role in Membrane Trafficking in *Caenorhabditis elegans*

**DOI:** 10.1371/journal.pone.0130778

**Published:** 2015-06-26

**Authors:** Ming Zhu, Gang Wu, Yu-Xin Li, Julia Kathrin Stevens, Chao-Xuan Fan, Anne Spang, Meng-Qiu Dong

**Affiliations:** 1 College of Life Sciences, Beijing Normal University, Beijing, China; 2 National Institute of Biological Sciences, Beijing, Beijing, China; 3 Peking University-Tsinghua University-National Institute of Biological Sciences Joint Graduate Program, National Institute of Biological Sciences, Beijing, Beijing, China; 4 Growth and Development, Biozentrum, University of Basel, Basel, Switzerland; NHLBI, NIH, UNITED STATES

## Abstract

The mammalian serum- and glucocorticoid-inducible kinase SGK1 regulates the endocytosis of ion channels. Here we report that in *C*. *elegans sgk-1* null mutants, GFP-tagged MIG-14/Wntless, the sorting receptor of Wnt, failed to localize to the basolateral membrane of intestinal cells; instead, it was mis-sorted to lysosomes. This effect can be explained in part by altered sphingolipid levels, because reducing glucosylceramide biosynthesis restored the localization of MIG-14::GFP. Membrane traffic was not perturbed in general, as no obvious morphological defects were detected for early endosomes, the Golgi apparatus, and the endoplasmic reticulum (ER) in *sgk-1* null animals. The recycling of MIG-14/Wntless through the Golgi might be partially responsible for the observed phenotype because the subcellular distribution of two plasma membrane cargoes that do not recycle through the trans-Golgi network (TGN) was affected to a lesser degree. Consistently, knockdown of the ArfGEF *gbf-1* altered the distribution of SGK-1 at the basolateral membrane of intestinal cells. In addition, we found that *sgk-1(RNAi) * induced unfolded protein response in the ER, suggesting at least an indirect role of SGK-1 early in the secretory pathway. We propose that SGK-1 function is required for lipid homeostasis and that it acts at different intracellular trafficking steps.

## Introduction

Mammalian serum- and glucocorticoid-inducible kinase 1 (SGK1) is an AGC kinase that was cloned as a gene whose transcription was stimulated by serum and glucocorticoids in rat mammary tumor cells [1–3]. Although SGK1 knockout mice display no severe defects [4–6], excessive expression of SGK1 leads to several disorders including hypertension, obesity, and tumor growth [4, 5]. In mammals, SGK1 is activated by insulin and growth factors through phosphoinositide 3-kinase (PI3-kinase) and 3-phosphoinositide (PIP3)-dependent kinase (PDK1) [5, 7]. SGK1 can be further activated by mammalian target of rapamycin complex 2 (mTORC2) [8]. Similar to another AGC kinase Akt (also called PKB), SGK1 can phosphorylate and inhibit the forkhead transcription factor FOXO3a (FKHRL1) [9]; but unlike Akt, SGK1 can activate nuclear factor-kappa B [10–12]. In response to a variety of stress stimuli, SGK1 up-regulates many ion channels, transporters and enzymes [13–15].

How SGK1 regulates these ion channels and transporters is mostly unknown. Recently, it was suggested that SGK1 is involved in the endocytosis of membrane proteins [16]. Cystic fibrosis transmembrane conductance regulator (CFTR) is a chloride channel residing on the apical plasma membrane (PM) of epithelial cells [17]. Curiously, while SGK1 inhibits the endocytosis of CFTR in human airway epithelial cells, it promotes the endocytosis of the epidermal growth factor receptor, which is also an apical plasma membrane protein [16]. Thus mammalian SGK1 may be involved in differentially regulating endocytosis of plasma membrane proteins.

Endocytosis is a key process by which cells internalize molecules [18]. Through receptor-mediated endocytosis, the major route in most cells, plasma membrane proteins and lipids are internalized in clathrin-coated vesicles and delivered to various destinations [19]. Once endocytosed, different cargoes are sorted in the early endosomes [20]: ligands typically enter the degradative pathway while their membrane receptors are often recycled back to the plasma membrane [21]. There are three different routes in which membrane receptors are recycled back to the plasma membrane: directly from sorting endosome through the tubular membrane structures (fast recycling), from the sorting endosome to the recycling endosomes or endosomal recycling compartments (ERC) (slow recycling), or through retrograde transport to the trans-Golgi network (TGN) followed by re-export to the plasma membrane [22–24].

In yeast, Ypk1—a homologue of SGK-1—activates serine palmitoyl-CoA acyltransferase (SPT) and promotes the biosynthesis of ceramide and sphingolipid [25, 26]. Ceramide is synthesized at ER and transported to the Golgi for conversion to sphingomyelin (SM) [27]. Ceramide is an important structural element of cell membranes and SM is one of the major lipid species in the lipid bilayer. Disruption of the biosynthesis of ceramide affects membrane trafficking [28].

In *C*. *elegans*, *sgk-1* encodes the sole ortholog of mammalian SGK1. Compared to the wild type (WT), *loss-of-function* (*lf*) mutants of *sgk-1* are abnormal in egg laying, development, stress response, and lifespan [29, 30], but the underlying mechanism is largely unknown. SGK-1 had been thought to regulate *C*. *elegans* lifespan in a way that resembles AKT-1 and AKT-2, by inhibiting the *C*. *elegans* FOXO transcription factor DAF-16 [29]. Recent genetic results suggested that SGK-1 activates DAF-16 [30–32]. However, it remains unknown whether *sgk-1* can regulate membrane trafficking in *C*. *elegans*.

## Materials and Methods

### *C. elegans* strains

Strains of *C*. *elegans* were cultured and maintained using standard protocols. The following strains or alleles were used: the wild-type N2, *pwIs765* (P_*vha-6*_MIG-14::GFP), *qxIs194* (P_*tat-1*_mCHERRY::MANS), *zcIs4* (*hsp-4pr*::*GFP*), *qxIs162* (P_*ges-1*_mCHERRY::TRAM), *qxEx2247* (P_*vha-6*_GLUT1::GFP), *pwIs112* (P_*vha-6*_hTAC::GFP), *pwIs846* (P_*vha-6*_tagRFP::RAB-5), *qxIs111* (P_*ges-1*_mCHERRY::RAB-7), and *pwIs72* (P_*vha-6*_GFP::RAB-5). *sgk-1(mg455)* was kindly provided by Alexander Soukas (Harvard Medical School). *sgk-1(ok538)* was obtained from CGC. *sgk-1(ok538)* and *sgk-1(mg455)* were backcrossed to N2 for 6 and 4 times, respectively, and assigned strain names MQD1027 and MQD1029. BR3063 *byEx* (P_*sgk-1*_SGK-1::GFP) was kindly provided by Ralf Baumeister (Albert-Ludwig University, Germany). GFP was fused to the C terminus of SGK-1, using a 7.3 kb genomic sequence containing the *a* isoform of *sgk-1* (5.1 kb) and a 2.2 kb upstream regulatory sequence. The sequence at the far 5’ end is: CTCCGGTAACTTACTCATTTTCAAC and the sequence at far 3’ end is: CGTCGACACCAATCGCGTTTTGGTC. BR3063 was integrated by gamma-irradiation and renamed as *hqIs150* (P_*sgk-1*_SGK-1::GFP). *hqIs150* was backcrossed to N2 for 3 times and assigned the strain name MQD862.

### Microscopy and imaging analysis

Differential interference contrast (DIC) and fluorescent images were captured with a Zeiss AxioImager M1 equipped with an AxioCam monochrome digital camera. A 10 × air Plan-Neofluar objective was used for detection of P_*hsp-4*_GFP. For confocal images, a Zeiss LSM 510 Meta inverted confocal microscope with 488 nm and 543 nm lasers was used, and images were processed and viewed using LSM Image Browser software and ZEN lite 2012 (Carl Zeiss). For SGK-1::GFP upon *gbf-1* RNAi, animals were mounted on 2% agarose pads in a drop of M9 containing 10 mM Levamisole, covered with a vaseline-rimmed cover slip and imaged with a spinning-disk confocal system Andor Revolution (Andor Technologies, Belfast, Northern Ireland) mounted onto an IX-81 inverted microscope (Olympus, Center Valley, PA) equipped with an iXon^EM^+ electron-multiplying charge-coupled device camera (Andor Technologies). Specimens were imaged using a 63×/1.42 numerical aperture oil objective. Each pixel represents 0.107 μm. Excitation was achieved using solid-state 488 nm laser. Exposure time was 100 ms.

### Quantitation of co-localization of mCHERRY::RAB-7 and MIG-14::GFP, the fluorescence intensity of MIG-14::GFP, hTAC::GFP, *hsp-4pr*::*GFP* and SGK-1::GFP with *gbf-1* RNAi

mCHERRY::RAB-7 positive ring-like vesicles containing 0, 1 or ≥ 2 MIG-14::GFP puncta were counted for six N2 worms and ten *sgk-1(ok538)* mutant worms, within a 3,036 μm^2^ area in the intestine of each worm. The cytoplasmic MIG-14::GFP was quantified by determining the average pixel intensity in non-overlapping 80 μm^2^ areas (8 μm × 10 μm) in the intestine (4 areas per worm, 8 worms of N2, 10 worms of *sgk-1(mg455)* and 13 worms of *sgk-1(ok538)*). The *P* value was calculated using Wilcoxon rank sum test. The cytoplasmic accumulation of hTAC::GFP was quantified by determining the average pixel intensity within 32 non-overlapping 80 μm^2^ areas (8 μm × 10 μm) in the intestine (4 areas per worm; 8 worms). The intensities of hTAC::GFP on the basal membrane and the lateral membrane were each measured as an average over 32 non-overlapping 35 μm^2^ areas (2.86 μm × 12.27 μm) in the intestine (4 areas per worm; 8 worms). The average pixel intensity per unit area was determined using Image J 1.46r software as described before [33]. The *P* value was calculated using Wilcoxon rank sum test. The expression of the *hsp-4* promoter driven GFP was quantified using Image J 1.46r software as follows. Each image containing 15 worms was taken using the same exposure time (1,000 ms). The background was subtracted by deleting pixels that are nearly completely black (grey values = 0, 1, or 2). Then, the average intensity of an entire image was measured. The results of 10 independent images are shown for each condition (a total of 150 worms). Z-stack images of SGK-1::GFP upon *gbf-1* RNAi were compressed in ImageJ version 1.48 (Wayne Rasband, National Institutes of Health, USA) using the average projection tool, and total fluorescence intensity and area was measured on the intestine that was outlined by the polygon selection tool.

### RNAi

RNAi was performed as described [34]. For RNAi feeding experiments, the full-length cDNA of *sgk-1* was cloned into the empty vector L4440, and the resulting plasmid pZM79 was used to transform the *E*. *coli* strain HT115. The *cgt-1*, *cgt-3*, *gbf-1* and *mon-2* RNAi bacterial strains were obtained from the Ahringer RNAi library. The *arl-1*, *cgt-1*, *cgt-3* and *unc-11* RNAi bacterial strains were obtained from the RCE1181 RNAi library. NGM plates containing 1 mM IPTG were inoculated with RNAi bacteria and induced for 12 hours at room temperature. Eggs were cultured at 20°C until L4. Bacteria transformed with the empty RNAi vector served as control for feeding. RNAi started from 5–10 adult worms for 3–4 days and the L4 larvae of the next generation were imaged. The only exception was the *gbf-1* RNAi experiment for SGK-1::GFP, in which RNAi started from L3 larvae for 3 days and the adult worms were imaged.

### Immunoprecipitation-Mass Spectrometry (IP-MS) analysis

Chromosomally integrated transgenic worms expressing SGK-1::GFP were cultured on high-growth (HG) plates. Lysates were made from 2 ml of packed, unsynchronized worms using FastPrep-24 (MP Biomedicals) in lysis buffer (20 mM Tris-HCl pH 8.0, 150 mM NaCl, 0.1% NP40, 2 mM EDTA, 1 × cocktail of protease inhibitors from Roche (Complete, EDTA free)). Proteins bound to the GBP (GFP binding protein) beads (ChromoTek) were eluted with 60 μl of 0.1 M glycine-HCl, pH 2.6, and neutralized with 10 μl of 1 M Tris-HCl, pH 8.0. The proteins were precipitated with 4 × volumes of cold acetone and then re-dissolved in 8 M urea, 100 mM Tris-HCl, pH 8.5. After reduction with 5 mM TCEP and then alkylation with 10 mM iodoacetamide, the samples were diluted fourfold to 2 M urea, 1 mM CaCl_2_, 20 mM methylamine, 100 mM Tris-HCl, pH 8.5, and digested with 1 μg of trypsin at 37°C overnight. The MS analysis was done in duplicates (two technical repeats). For each technical repeat, one fourth of the resulting peptides were pressure-loaded onto a fused silica capillary column packed with 5 μm Luna C18 material (RP, Phenomenex, Ventura, CA), with a Kasil frit at the end. The column was washed with a buffer containing 95% water, 5% acetonitrile, and 0.1% formic acid. After desalting, a 9 cm 100 μm i.d. capillary with a 5 μm pulled tip packed with 5 μm Luna C18 material was attached to the two-phase column with a union. The peptides were separated over a 2-h reverse phase gradient generated by an Agilent 1100 quaternary HPLC (Agilent) and sprayed directly into an LTQ Orbitrap mass spectrometer (ThermoFisher Scientific) with the application of a distal 2.5 kV spray voltage. The gradient was as follows: 5 min from 100% buffer A (5% acetonitrile, 0.1% formic acid) to 5% buffer B (80% acetonitrile, 0.1% formic acid), then increasing to 30% buffer B over 75 min, further to 80% buffer B in 10 min, to 100% buffer B in 10 min, and lastly, a 20-min 100% buffer B wash. The flow rate was maintained at 0.1 ml/min and the flow was split using a microTee that was connected, at one of its openings, to an empty 50 μm i.d. capillary. The length of the empty capillary was adjusted so that a flow rate of about 200 nl/min was achieved at the tip of the column. The mass spectrometer was operated in the data-dependent mode. Survey MS scans were acquired in the orbitrap with the resolution set to a value of 60000. Each survey scan (400−2000 m/z) was followed by 8 data-dependent tandem mass (MS/MS) scans in the linear ion trap at 35% normalized collision energy. AGC target values were 200,000 for the survey scan and 10,000 for the MS/MS scan. Target ions already selected for MS/MS were dynamically excluded for 30 seconds. Proteins were identified by searching the MS/MS spectra against a *C*. *elegans* protein database using Prolucid [35] and filtering the search results with DTASelect 2.0 [36] at 0.01 false discovery rate (FDR) at the spectral level, 5 ppm precursor mass accuracy, and a minimal Z score of 4.0. The overall false discovery rate for protein identification is less than 0.5%. Using the control IP-MS results of seventeen transgenic strains expressing unrelated GFP fusion proteins, identified proteins were ranked by WD scores calculated as described previously [37]. WD scores help downgrade common contaminant proteins and enrich for specific binding proteins [37]. The RAW data of SGK-1::GFP IP-MS experiments has been deposited to the ProteomeXchange Consortium [38] via the PRIDE partner repository with the dataset identifier PXD002190.

### Yeast two-hybrid analysis

We used the Matchmaker system (Clontech). The cDNA of the *sgk-1* gene was cloned into a prey vector modified from the pGAD GH vector (Clontech). The cDNA of the *arl-1*, *unc-11*, *cogc-3* and fragments of *mon-2*, *gbf-1* were cloned into a bait vector modified from the pGBKT7 vector (Clontech). Bait and prey plasmids were co-transformed into the AH109 strain and transformants were selected on the double dropout medium (SD/–Leu/–Trp). The activation of the HIS3 reporter gene was assessed on the triple dropout medium (SD/–Leu/–Trp/-His).

### Quantitative RT-PCR

Total RNA was extracted from synchronized L4 worms using TRIZOL (Invitrogen), followed by the removal of contaminant DNA using DNase I. cDNAs were synthesized from the total RNA templates using a reverse transcription kit (Takara). Primers used for qPCR of *hsp-4* were primers #1 [GTGGCAAACGCGTACTGTGATGA]/[CGCAACGTATGATGGAGTGATTCT], primers #2 [TTCCGTGCTACATTGAAGCCGGTT]/[GCTTCGTCAGGGTTGATTCCACGA], primers #3 [GGACTTGTTCCGTGCTACATTGAAG]/[GCTTCGTCAGGGTTGATTCCACGA] and pmp-3F [GAATGGAATTGTTTCACGGAATGC]/ pmp-3R [CTCTTCGTGAAGTTCCATAACACGATG] for *pmp-3* as the internal standard. qPCR was carried out on an ABI 7500 Fast real-time PCR system using a Takara real-time PCR kit (SYBR Premix Ex TaqTM II).

### Feeding of Myriocin

A 1 mg/ml myriocin (Sigma) stock solution was made in methanol. Gravid adults (five per plate) were transferred to plates containing UV-irradiated (10 min at 100 mJ/cm^2^) OP50 mixed with an equal volume of myriocin or solvent control (methanol). Precipitation of myriocin occurred at 25.2 μM and 50.4 μM. The first batch of L4 larvae of the next generation were imaged.

## Results

### In *sgk-1* mutants MIG-14 failed to localize to the basolateral membrane of intestinal cells

To determine whether SGK-1 plays a conserved role in endocytosis, we investigated the function of SGK-1 in recycling of the plasma membrane protein MIG-14. *mig-14* encodes the *C*. *elegans* homologue of Wntless, an evolutionarily conserved multi-pass transmembrane protein, which binds Wnt in the Golgi and escorts Wnt to the plasma membrane for its release. Wnt proteins are a conserved family of secreted lipid-modified signaling glycoproteins that play a critical role in embryonic development [39]. Plasma membrane-bound MIG-14 is retrieved through endocytosis and transported to the Golgi in a retromer-dependent manner. In mutant *C*. *elegans* lacking subunits of the retromer complex, MIG-14 is mis-sorted into late endosomes and lysosomes [40–42].

**Fig 1:**
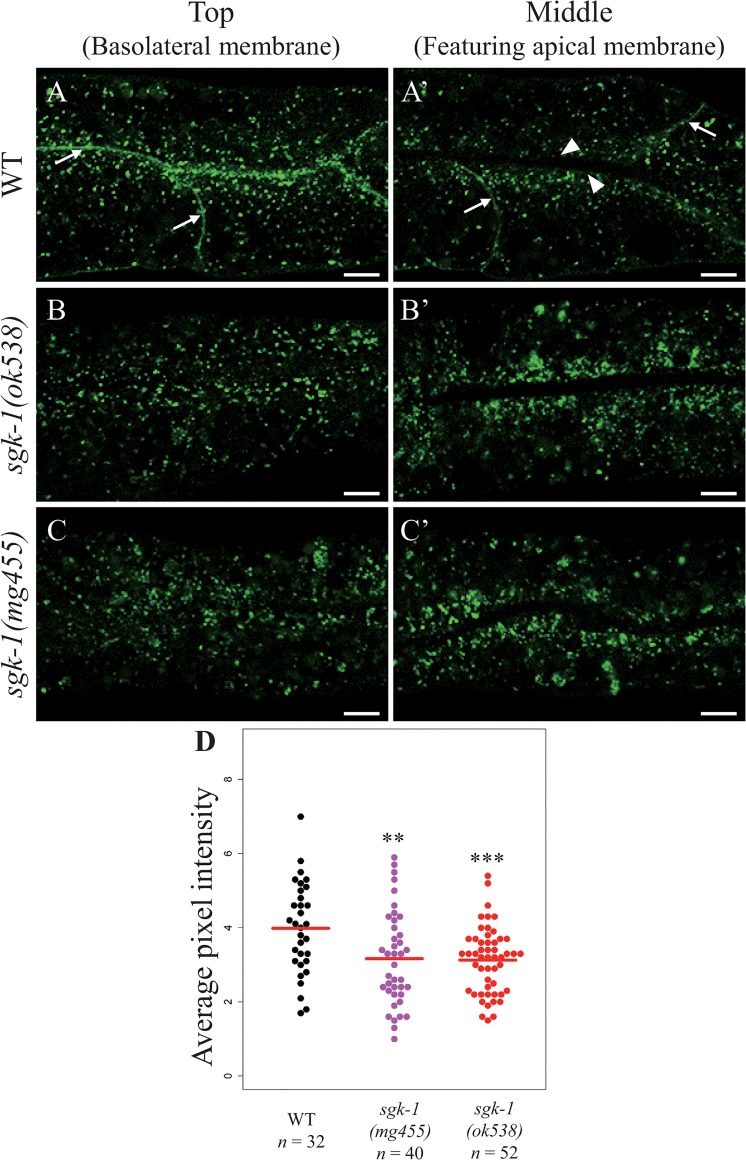
Both putative sgk-1(null) alleles abolished the plasma membrane localization of MIG-14::GFP in intestinal cells. Confocal images of the intestine of wild type (A, A’), *sgk-1(ok538)* (B, B’) and *sgk-1(mg455)* (C, C’) animals expressing MIG-14::GFP. In wild type intestinal cells, MIG-14::GFP is seen on the basolateral membrane (arrows, best viewed on a focal plane near the top of the cell) but not on the apical membrane (arrowheads, best viewed on a focal plane in the middle of the cell). Scale bars: 5 μm. (D) Quantitation of average pixel intensity of MIG-14::GFP localized in the cytoplasm. The average intensity is denoted with a red line. *** *P* value <0.001, ** *P* value <0.01 (Wilcoxon rank sum test).

The distribution of MIG-14/Wntless clearly changed in *sgk-1* mutants. In wild-type animals, MIG-14::GFP is located on the basolateral membrane (Fig 1A) and also on dispersed punctate structures in the cytoplasm (Fig 1A’). In two putative null mutants of *sgk-1*, *ok538* and *mg455*, the basolateral membrane localization of MIG-14::GFP was hardly detectable, and most of MIG-14::GFP punctate structures localized near the apical membrane and the averaged fluorescence intensity of the cytoplasmic MIG-14::GFP decreased slightly (Fig 1B–1D and S1–S5 Figs). Thus the subcellular localization of MIG-14 is dependent on SGK-1.

### MIG-14::GFP was mis-sorted into early lysosomes in *sgk-1* mutant worms

**Fig 2:**
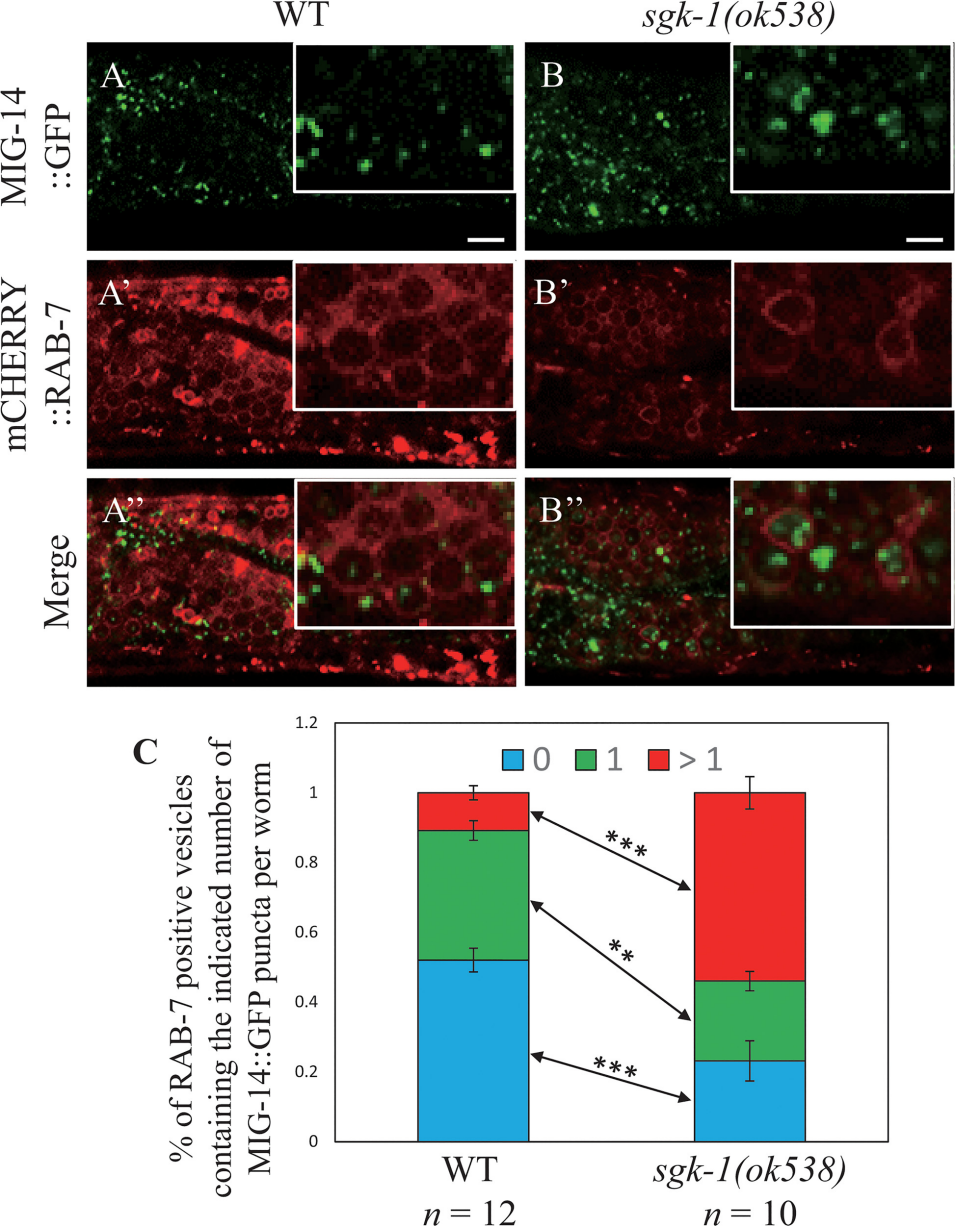
MIG-14::GFP was delivered to early lysosomes marked by RAB-7. Confocal images of the wild type (A, A’, A”) and *sgk-1(null)* (B, B’, B”) intestinal cells expressing MIG-14::GFP (A, B) or mCHERRY::RAB-7 (A’, B’). Insets show magnified areas (× 2.5). Scale bars: 5 μm. Quantitation of RAB-7-positive ring-like vesicles with or without MIG-14::GFP puncta inside (C). *** *P* value <0.001, ** *P* value <0.01 (Student’s *t*-test).

Cargo that cannot be retrieved to the TGN remains mostly in endosomes and hence should be transported to lysosomes [42]. To test this notion, we determined the subcellular localization of MIG-14::GFP in *sgk-1* mutants. In wild-type animals, late endosomes and early lysosomes are both marked by mCHERRY::RAB-7 but can be distinguished morphologically: the former are punctate structures and the latter ring-like vesicles [33, 43]. We found that mCHERRY::RAB-7 labeled ring-like vesicles contained no or only a single MIG-14::GFP punctum in the wild type (Fig 2, panels A, A’, A” and C), while most of the them contained two or more MIG-14::GFP puncta in the *sgk-1* mutant, indicating that MIG-14::GFP is mis-sorted to lysosomes (Fig 2, panels B, B’, B” and C). A similar phenotype of MIG-14::GFP was reported in a mutant lacking *rme-8* [42], which encodes a retromer-associated protein.

### Golgi-independent recycling of membrane receptors was defective in *sgk-1* mutants

**Fig 3:**
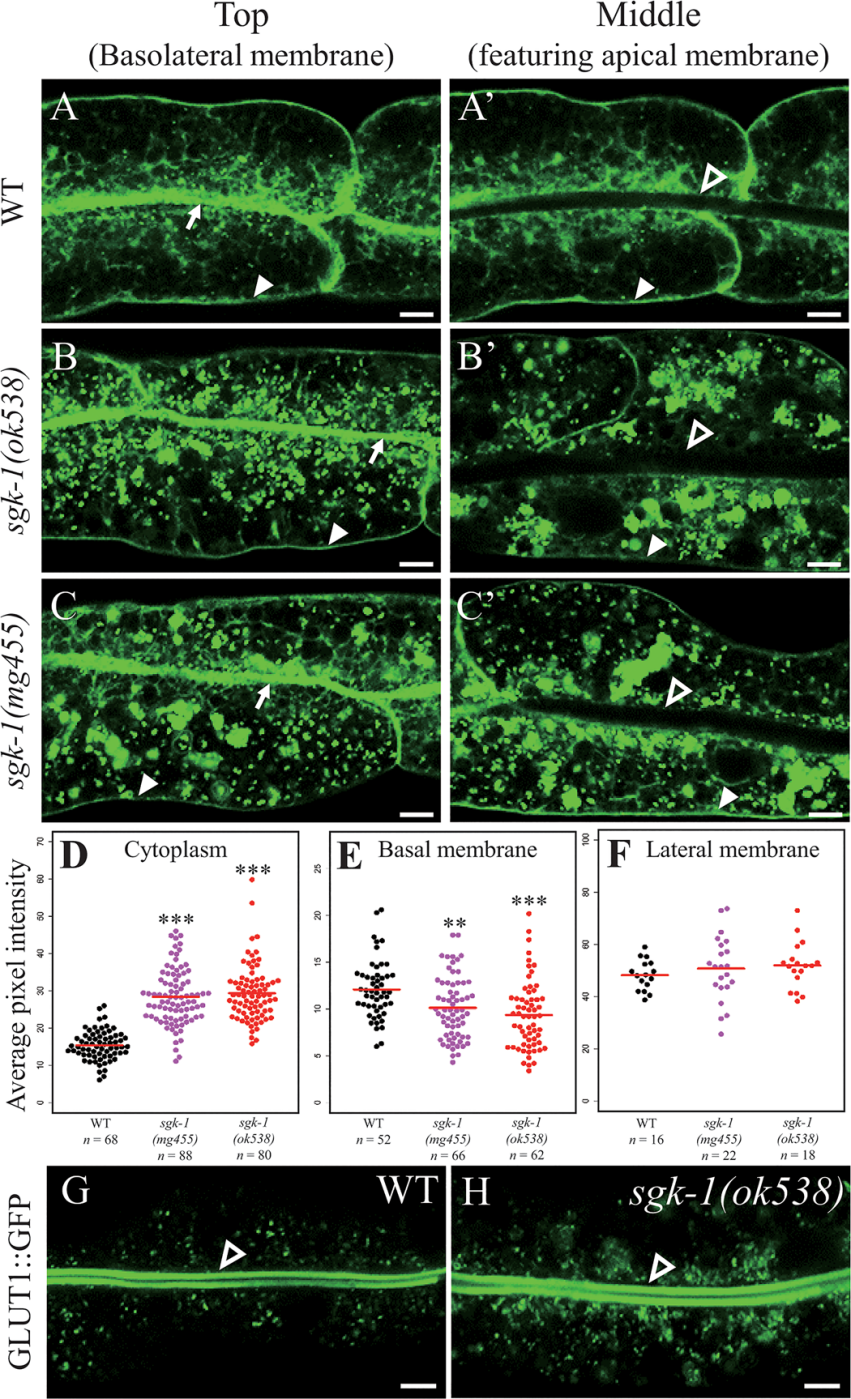
Defective membrane trafficking of hTAC::GFP and GLUT1::GFP in *sgk-1(null)* mutants. Confocal images of wild type (A, A’, G), *sgk-1(ok538)* (B, B’, H) and *sgk-1(mg455)* (C, C’) intestinal cells expressing hTAC::GFP or GLUT1::GFP. The apical, basal and lateral membranes are indicated by open arrow heads, solid arrow heads and arrows, respectively. Scale bars: 5 μm. See Fig 2 legend for explanations of different focal planes. (D-F) Quantitation of average pixel intensity of hTAC::GFP localized in the cytoplasm (D), on the basal membrane (E) or lateral membrane (F). The average intensity is denoted with a red line. *** *P* value <0.001, ** *P* value <0.01 (Wilcoxon rank sum test).

Given that we only investigated a cargo that recycles through the Golgi so far, we asked next whether recycling to the plasma membrane was generally affected in *sgk-1* mutant animals. hTAC (the a-chain of the human IL-2 receptor TAC) and GLUT1 (glucose transporter 1) are plasma membrane receptors internalized via clathrin-independent endocytosis and recycled back to the plasma membrane through the endocytic recycling compartment (ERC) and RME-1-positive basolateral recycling endosomes [44, 45]. hTAC::GFP mainly localized to the basolateral membrane in the wild type (Fig 3, panels A and A’). In the *sgk-1(ok538)* mutant, the pool of hTAC::GFP on the basolateral membrane decreased slightly. However, strikingly, large hTAC::GFP structures, possibly aggregated vesicles, accumulated in the cytoplasm (Fig 3, panels B, B’ and D). In the *sgk-1(mg455)* mutant, similar cytoplasmic accumulation of hTAC::GFP was observed, as well as a slight reduction of hTAC::GFP on the basal membrane (Fig 3, panels C, C’, D, E and F, and S1, S2 and S6–S8 Figs). To further explore whether the internal aggregations are abnormal early endosomes, we determined the distribution of hTAC::GFP and an early endosome marker, RFP::RAB-5 in wild type and *sgk-1(ok538)* mutant worms. In wild type worms, only a small portion of the cytoplasmic hTAC::GFP puncta co-localized with early endosomes labeled by RFP::RAB-5 (S9 Fig) In *sgk-1(ok538)* mutant worms, most of the hTAC::GFP labeled large, aggregated structures that are also labeled by RFP::RAB-5 (S9 Fig). It is unclear whether these structures were abnormal early endosomes or protein aggregates that contain both hTAC::GFP and RFP::RAB-5.

In intestinal cells of L4 larvae, GLUT1::GFP is limited to the apical membrane and punctate structures near the apical membrane (Fig 3G). Loss of *sgk-1* did not affect the apical membrane localization of GLUT1::GFP, but it increased the amount of cytosolic GLUT1::GFP near and away from the apical membrane (Fig 3H). In young wild-type adults, GLUT1::GFP is seen on both apical and basolateral membranes [43]. We found that the basolateral membrane localization of GLUT1::GFP was abolished in young *sgk-1* mutant adults (S10 Fig). The reason for the change in localization during development is unclear.

The above results suggest that *sgk-1* plays a role in intracellular traffic of plasma membrane receptors. The less pronounced phenotype observed for GLUT1::GFP may suggest that apical recycling is less profoundly dependent on SGK-1 than basolateral recycling.

### Reduction of glucosylceramide alleviates the *sgk-1(lf)* induced trafficking defects

**Fig 4:**
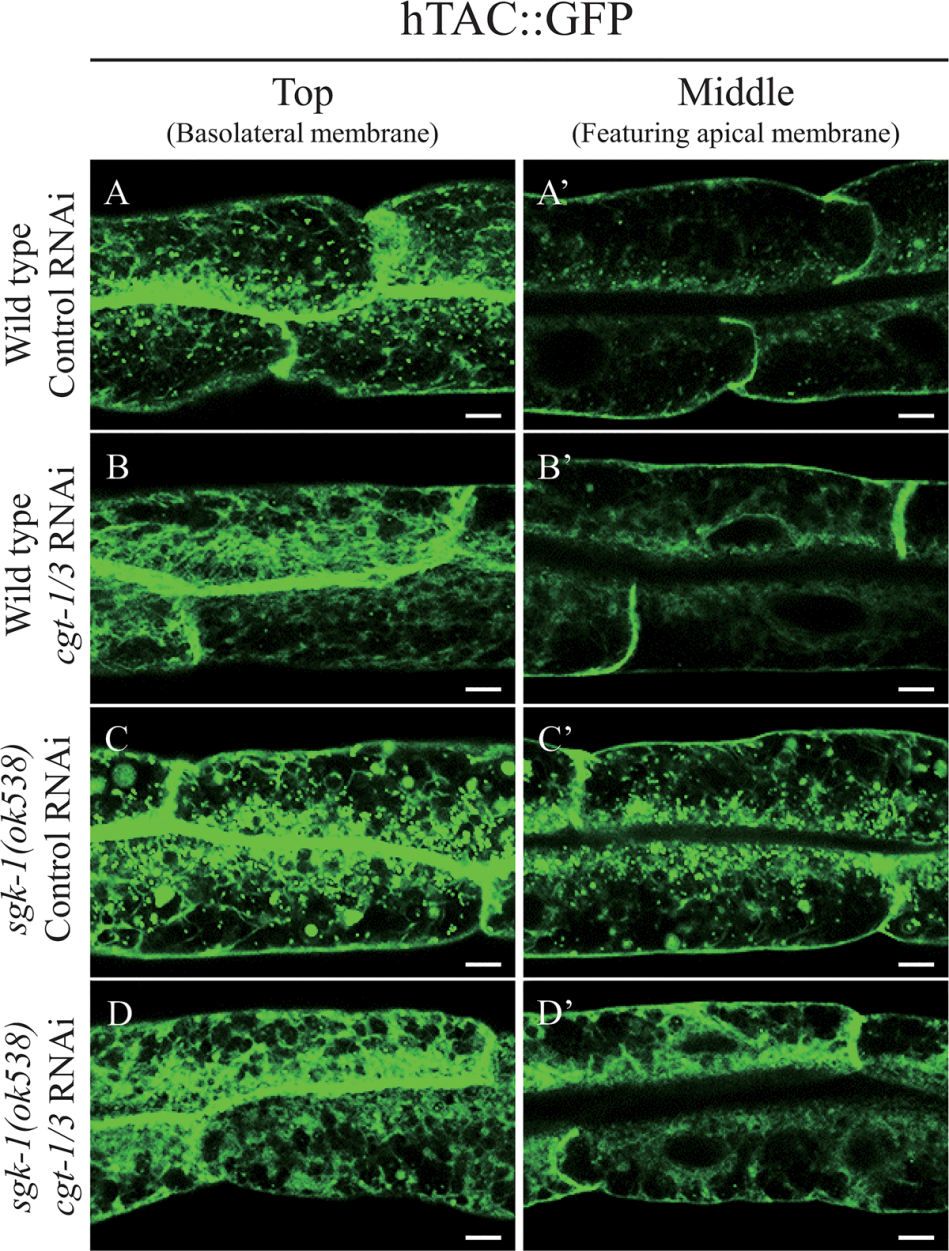
The effect of *cgt-1/3* double RNAi on the localization patterns of hTAC::GFP. Confocal images of intestinal cells of wild type worms treated with control RNAi (A, A’), *cgt-1/3* double RNAi (B, B’) and *sgk-1(ok538)* mutant animals treated with control RNAi (C, C’), *cgt-1/3* double RNAi (D, D’) expressing hTAC::GFP. The basolateral membranes were indicated by arrows. Scale bars: 5 μm.

**Fig 5:**
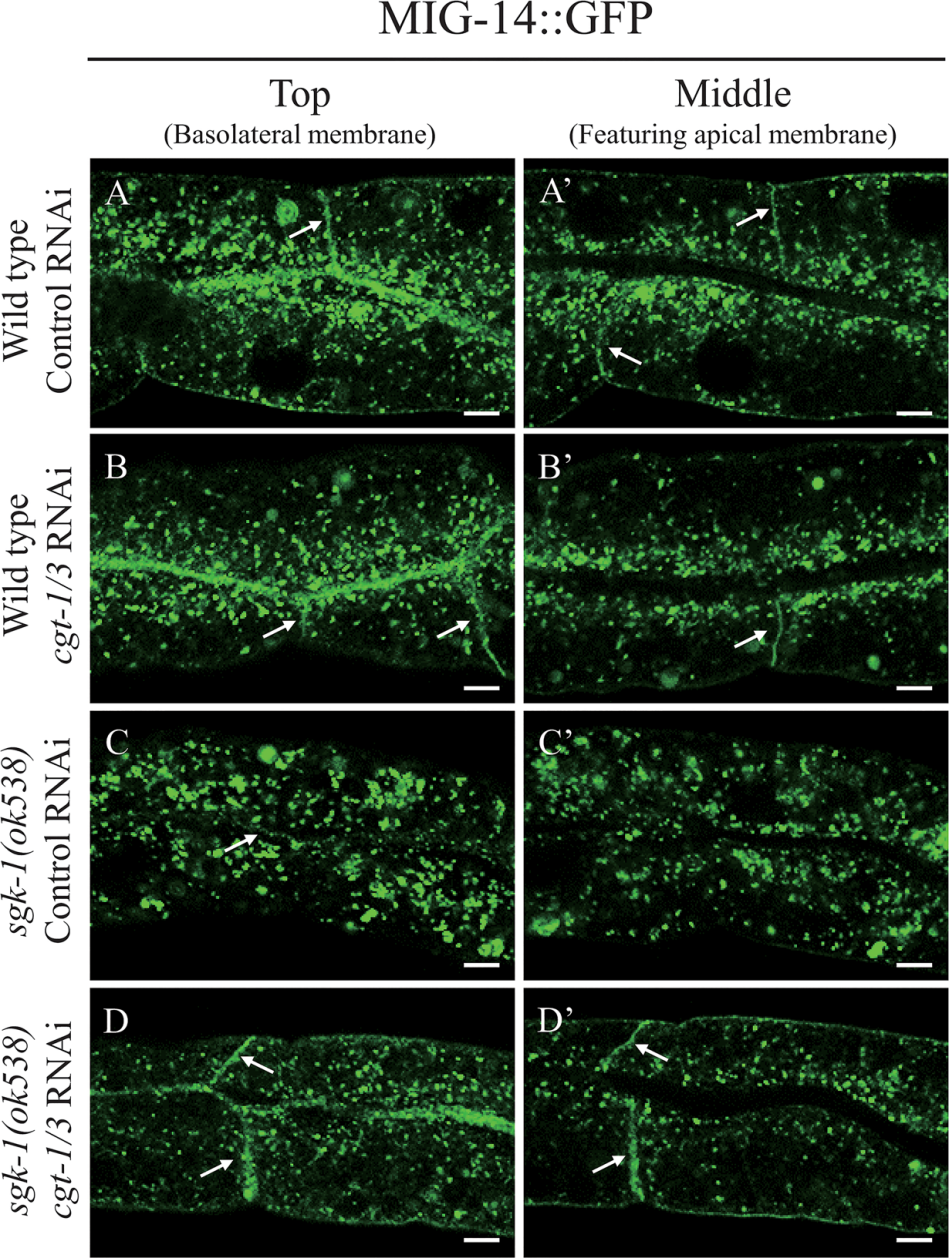
The effect of *cgt-1/3* double RNAi on the localization patterns of MIG-14::GFP. Confocal images of intestinal cells of wild type worms treated with control RNAi (A, A’), *cgt-1/3* double RNAi (B, B’) and *sgk-1(ok538)* mutant animals treated with control RNAi (C, C’), *cgt-1/3* double RNAi (D, D’) expressing MIG-14::GFP. The basolateral membranes were indicated by arrows. Scale bars: 5 μm.

**Fig 6:**
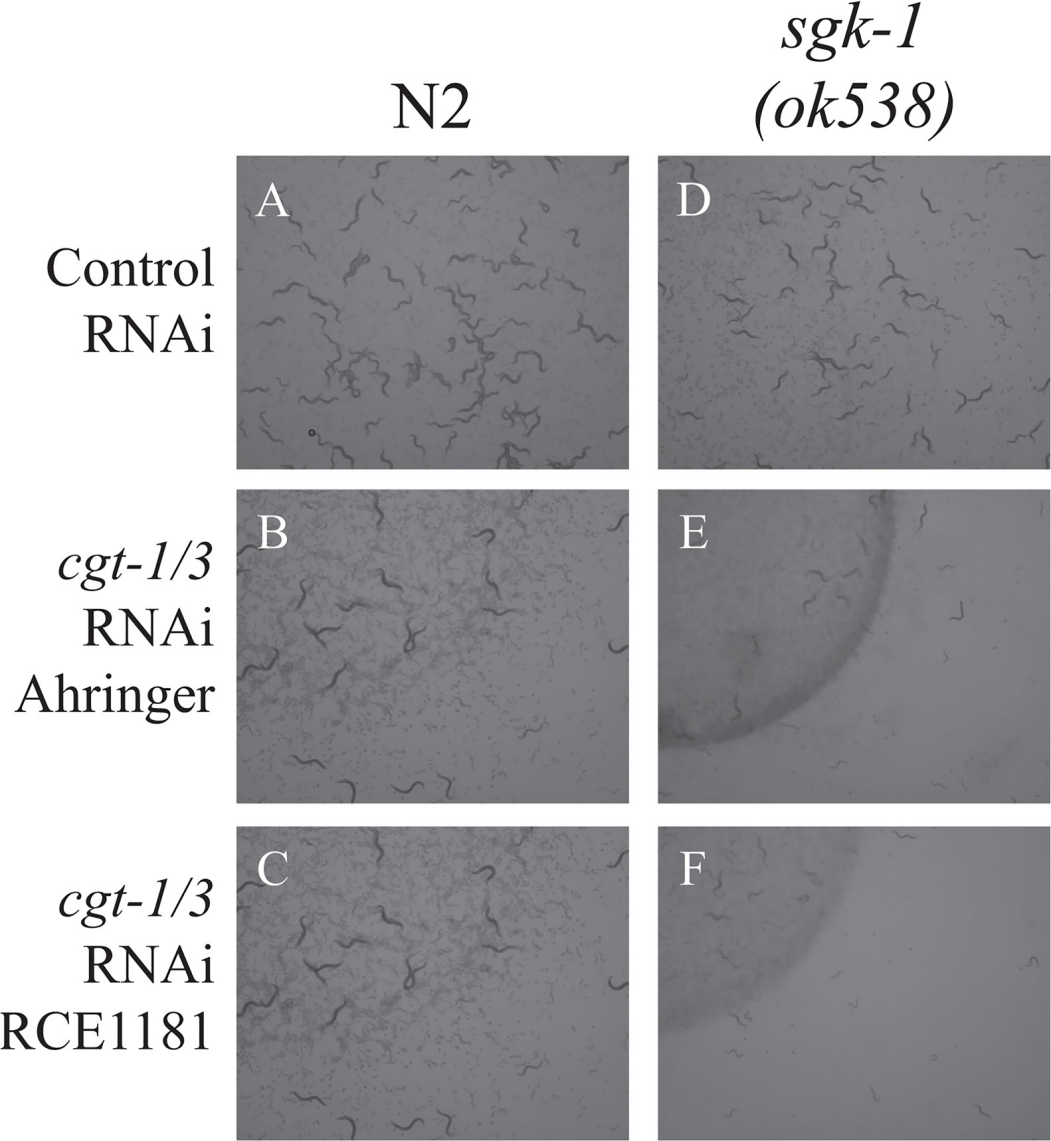
*sgk-1* mutant worms treated with *cgt-1/3* double RNAi developed very slowly. Images of wild type (A, B, C) and *sgk-1(ok538)* mutant animals (D, E, F) treated with control RNAi (A, D) and *cgt-1/3* double RNAi from Ahringer library (B, E) and RCE1181 library (C, F). The experiments started with 5 gravid adult worms on each plate and the plates were imaged 5 days later.

To find out whether *sgk-1* regulates membrane trafficking through sphingolipids, we first tested Myriocin, an inhibitor of the first and the key step in sphingosine biosynthesis. We fed the wild type and *sgk-1(ok538)* worms with 4.2 μM, 25.2 μM and 50.4 μM of Myriocin, but did not detect any obvious growth or morphological effect in either strain. Also, we did not see any effect of Myriocin on the localization pattern of either hTAC::GFP or MIG-14::GFP in wild type animals (S11 and S12 Figs). It is plausible that the drug may not accumulate efficiently in cells to show an effect. Therefore, we knocked-down *cgt-1* and *cgt-3*, which encode two of the three ceramide glucosyltransferases that are important for the synthesis of glucosylceramide and glycosphingolipids. Similar to the *cgt-1; cgt-2; cgt-3* triple mutant, *cgt-1; cgt-3* double mutant animals have reduced glucosylceramide levels and arrest at the L1 larval stage [46]. In about 50% of the wild-type worms, *cgt-1/3* double RNAi reduced the expression of hTAC::GFP to an undetectable level. In the remaining 50% wild-type worms, *cgt-1/3* RNAi significantly decreased the amount of small cytoplasmic hTAC::GFP puncta (Fig 4). Larger-sized hTAC::GFP puncta or aggregates were seen in the cytoplasm of intestinal cells in the *sgk-1* mutant, but they were reduced in number upon *cgt-1/3* RNAi (Fig 4). Therefore, *cgt-1/3* RNAi alleviated the phenotype of *sgk-1* null with respect to hTAC::GFP. Similarly, *cgt-1/3* double RNAi suppressed *sgk-1* with respect to MIG-14::GFP. *sgk-1* null nearly abolished MIG-14::GFP on the basolateral membrane, but this was restored by *cgt-1/3* double RNAi (Fig 5). It has been reported that worms treated with *cgt-1/3* double RNAi arrested at the L1 stage [46], but in our hands, only some of the *cgt-1/3* double RNAi animals arrested at L1, probably due to a weaker RNAi effect. The *sgk-1* mutant worms develop somewhat more slowly than the wild type. Interestingly, *sgk-1* mutant worms treated with *cgt-1/3* double RNAi developed very slowly (Fig 6). Altogether, our results show that *sgk-1* and *cgt-1/3* have complex genetic interactions. Our data indicate that SGK-1 plays a conserved role in lipid homeostasis.

### *sgk-1(lf)* did not affect the morphology of early endosomes, the Golgi and ER

**Fig 7:**
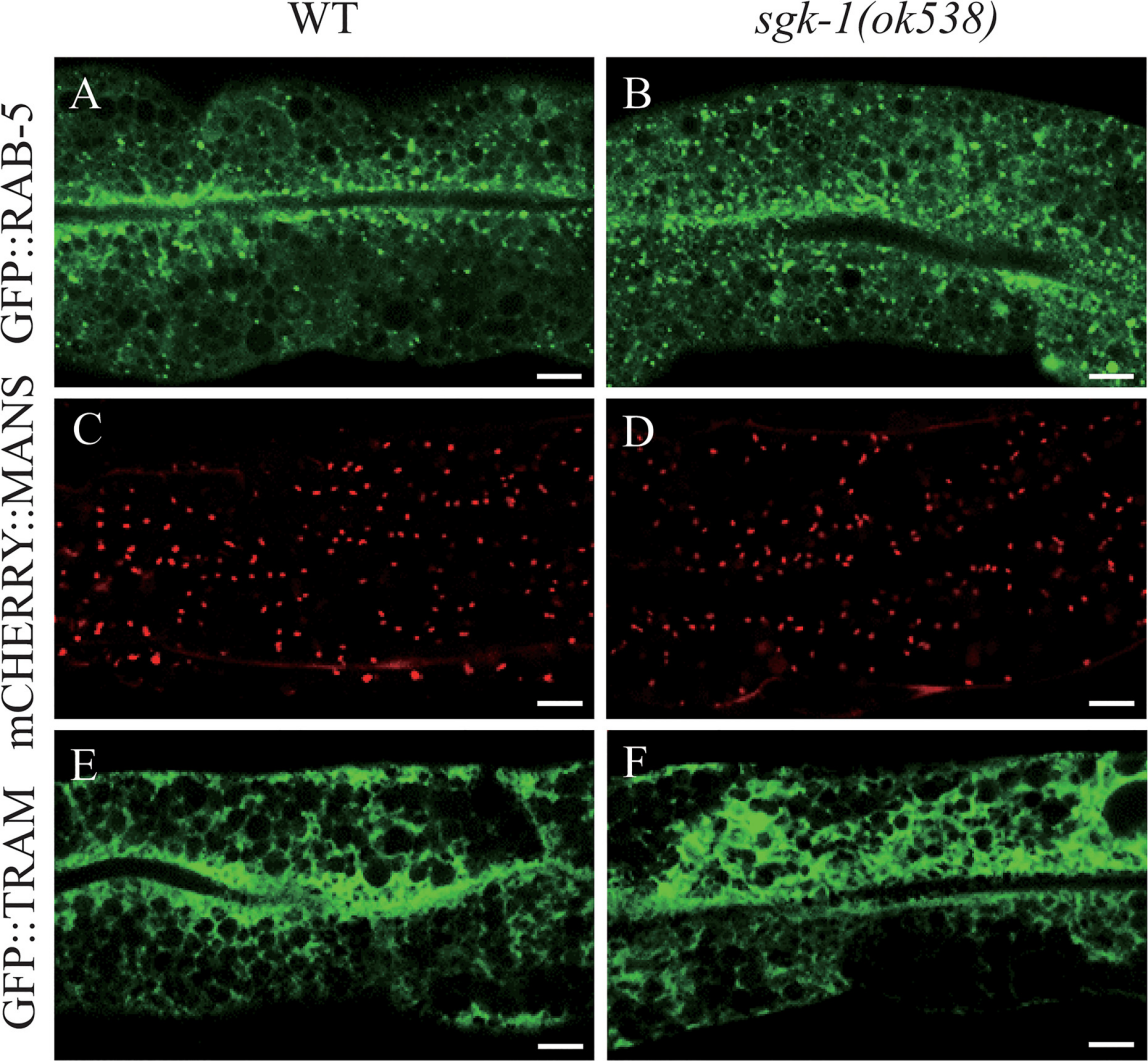
Wild type-like morphology of early endosomes, the Golgi and ER in *sgk-1(lf)* mutant. Confocal images of the wild type (A, C, E) and *sgk-1(ok538)* (B, D, F) intestinal cells expressing GFP::RAB-5 (A, B), mCHERRY::MANS (C, D) or GFP::TRAM (E, F). Scale bars: 5 μm.

**Fig 8:**
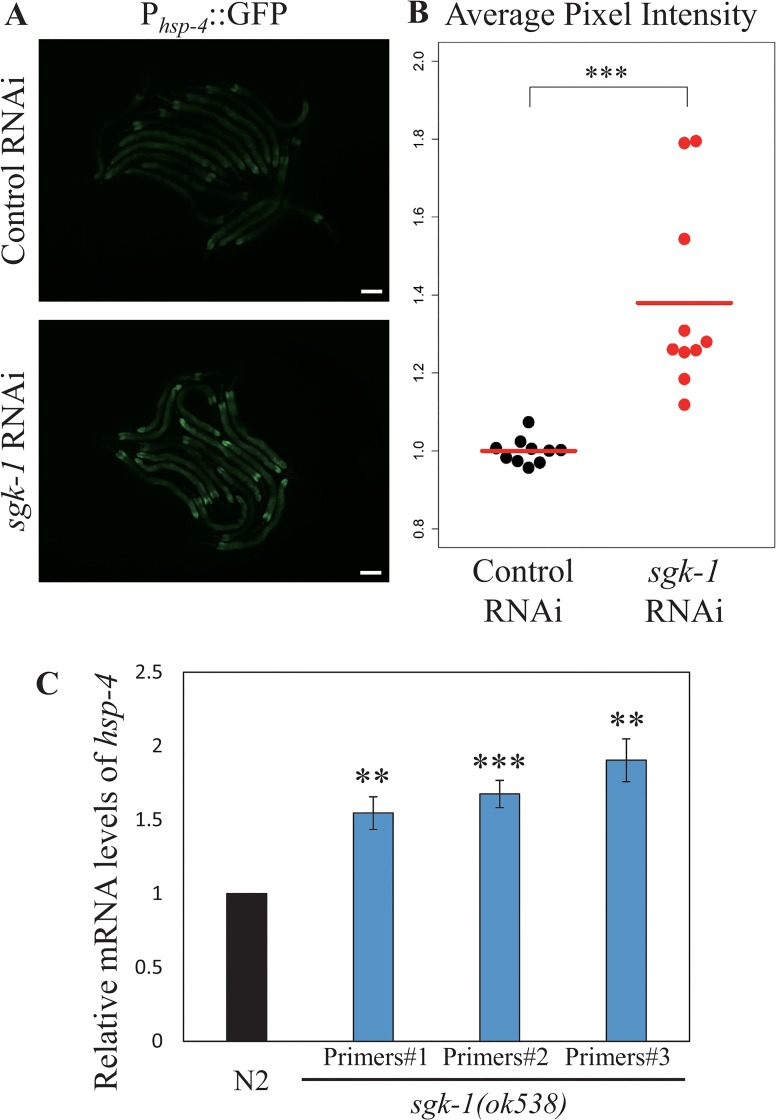
Loss of *sgk-1* activity induced the UPR-ER. (A) Fluorescent images of worms carrying a GFP transgene under the *hsp-4* promoter and treated with control RNAi (empty vector pL4440) or *sgk-1* RNAi. Scale bars: 50 μm. (B) Quantitation of average pixel intensity of the images shown in (A) and 18 additional images, nine for control RNAi and nine for the *sgk-1* RNAi. The average intensity is denoted with a red line. There were fifteen L4 worms in each image and ten images for each treatment. *** *P* value <0.001 (Student’s *t*-test). (C) Relative mRNA levels of *hsp-4* in wild type and *sgk-1(ok538)* mutant animals. ** *P* value <0.01, * *P* value <0.05 (Student’s t-test).

So far we provided evidence that plasma membrane proteins are mis-localized in *sgk-1* mutants. One explanation for the phenotype is that the proteins do not reach the plasma membrane efficiently. Therefore, we asked next, whether the morphology of the organelles along the secretory pathway were affected in *sgk-1* mutant animals. First, we examined early endosomes by checking the distribution of GFP::RAB-5 in the wild type and *sgk-1(ok538)* backgrounds (Fig 7, panels A and B). No obvious difference was detected, suggesting that SGK-1 function is not required for the formation of early endosomes. In earlier experiments we observed large aggregates in the *sgk-1(ok538)* mutant that were positive for RFP::RAB-5 and hTAC::GFP (S9 Fig), but it was unclear whether these were protein aggregates or abnormal early endosomes. Given that *sgk-1(ok538)* had no effect on GFP::RAB-5, they were most likely protein aggregates that contained both hTAC::GFP and RFP::RAB-5. Alternatively, loss of *sgk-1* may affect early endosomes only in a sensitized background such as when the trafficking system is overloaded with cargoes. Similarly, loss of *sgk-1* did not change the morphology of the Golgi compartment labeled by mCHERRY::MANS (mannosidase), which appeared as dispersed punctate structures in the intestinal cells (Fig 7, panels C and D). Next, we examined the morphology of ER using a GFP-tagged TRAM (translocating chain-associating membrane protein), an ER-specific marker, and found no obvious difference between wild type and the *sgk-1* mutant (Fig 7, panels E and F). However, loss of *sgk-1* induced unfolded protein response in the ER (UPR-ER) as indicated by an elevated expression of *hsp-4pr*::*GFP* (Fig 8, panels A and B). We confirmed the UPR induction by detecting increased *hsp-4* mRNA levels in *sgk-1(ok538)* L4 mutant worms (Fig 8C). Together, these results indicate that the morphology of early endosomes, Golgi and ER is not grossly altered and that *sgk-1* may be important for protein homeostasis.

### SGK-1::GFP is distributed at the cortex of the plasma membrane and throughout the cytoplasm of intestinal cells

**Fig 9:**
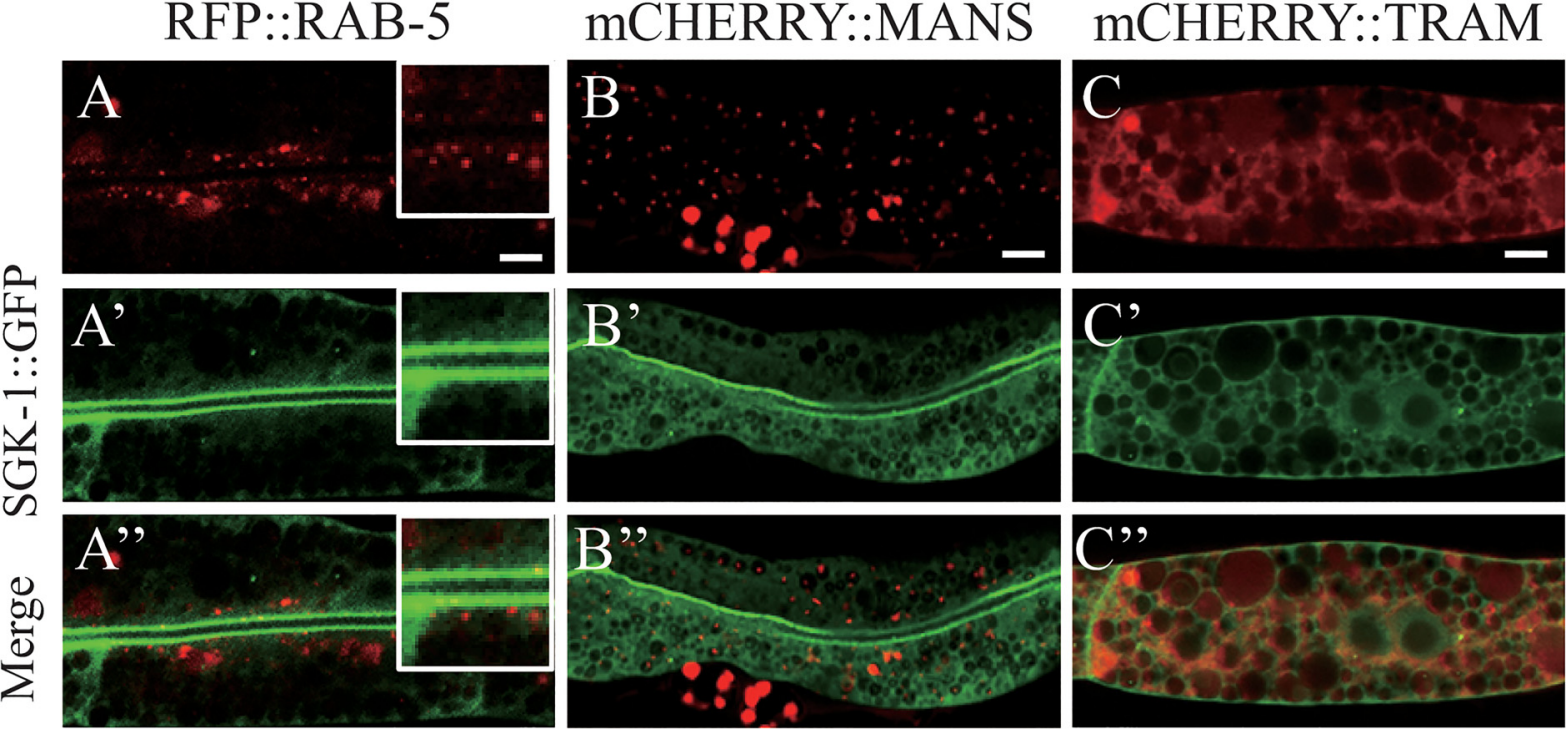
SGK-1::GFP did not colocalize with RFP::RAB-5, mCHERRY::MANS or mCHERRY::TRAM. Confocal images of the intestine in transgenic worms expressing SGK-1::GFP together with RFP::RAB-5 (A-A”), mCHERRY::MANS (B-B”) or mCHERRY::TRAM (C-C”). Insets show magnified areas (× 2). Scale bars: 5 μm.

To find out where SGK-1 may physically interact with the membrane trafficking system, we examined the distribution of SGK-1::GFP driven by its own promoter. SGK-1::GFP was expressed at a high level in the intestine, as well as the head and tail neurons (S13 Fig). In the intestinal cells, SGK-1::GFP was seen at the cortex of the plasma membrane (most prominently the apical membrane) (Fig 9A’ and S13 Fig) and throughout the cytoplasm in a diffuse pattern (Fig 9, panels B’ and C’, and S13B Fig), occasionally in punctate structures over a diffuse background (not shown). The cortical SGK-1::GFP pool did not colocalize with RFP::RAB-5 (Fig 9, panels A, A’ and A”). The diffused appearance of SGK-1::GFP in the cytoplasm precludes the conclusion of co-localization of SGK-1::GFP and mCHERRY::MANS or mCHERRY::TRAM, an ER marker (Fig 9). However, it is possible that SGK-1 may interact with membrane proteins or membrane-associated proteins and is transiently recruited to organelles.

### SGK-1 interacts with proteins involved in the regulation of intracellular transport

To identify potential SGK-1 binding proteins on membranes, we performed co-immunoprecipitation experiments. Using GFP binding protein (GBP), which is a high-affinity single-chain antibody of GFP [47], we immunoprecipitated (IP) a SGK-1::GFP fusion protein expressed in *C*. *elegans* under its own promoter. Co-immunoprecipitated proteins were identified using mass spectrometry (MS). After subtracting background binding proteins and non-specific interacting proteins that co-immunoprecipitated with GFP alone or a number of unrelated GFP fusion proteins [37], we found five SGK-1 associated proteins with an established function in intracellular transport: ARL-1, COGC-3, GBF-1, MON-2 and UNC-11 (Table 1). Interestingly, homologs of all but UNC-11 have been reported to be localized to the Golgi apparatus [48–52]. For example, the human homologue of ARL-1 is localized to the Golgi and is a member of the Arf/Sar family small GTPases, which are activated by guanine nucleotide exchange factors (ArfGEFs) [48, 49]. By sequence similarity, GBF-1 and MON-2 are classified as ArfGEFs and both are shown to reside on the Golgi, although MON-2 lacks a domain responsible for guanine nucleotide exchange [50, 52]. In addition, COGC-3 is part of a tethering complex at the Golgi [51].

**Table 1:** Proteins co-immunoprecipitated with SGK-1 are involved in membrane trafficking.

Protein Name	Spectral count of Rep 1 and 2		Mass Spec Identification of Membrane Trafficking Proteins Co-immunoprecipitated with SGK-1
ARL-1	2	3	Homologous to human ARF1, a small GTPase that localizes to the Golgi apparatus and plays a central role in intra-Golgi transport [48].
COGC-3	1	1	Ortholog of mammalian COG-3/Sec34, a subunit of lobe A of the conserved oligomeric Golgi complex (COGC) [51].
GBF-1	1	4	Guanine nucleotide exchange factor of the Arf family small GTPases (ArfGEF). GBF-1 localizes to the cis-Golgi, is part of the t-ER-Golgi elements, required for secretion and the integrity of the Golgi and ER. [50]
MON-2	3	3	ArfGEF-like protein orthologous to *Saccharomyces cerevisiae* Mon2p and the vertebrate MON2 proteins, predicted to function in the endosome-to-Golgi retrograde transport in *C*. *elegans* [52].
UNC-11	1	2	clathrin-adaptor protein AP180 that functions in clathrin-mediated endocytosis [53].

Spectral count: number of spectra that identify a protein, suggestive of the abundance of this protein in the sample. The spectral counts of SGK-1 are 302 and 412 in two technical repeats (Rep 1 and Rep 2). The spectral counts of the five membrane trafficking proteins are low, indicating their low abundance in the SGK-1::GFP immunoprecipitates. However, these proteins were highly specific for SGK-1::GFP, for they were not seen in the immunoprecipitates of seventeen other unrelated GFP fusion proteins (not shown).

MON-2 and COGC-3 also interacted with SGK-1 in yeast two-hybrid assay, validating these two hits (S14 Fig). To find out whether SGK-1 regulates membrane trafficking through interaction with our candidate proteins, we assessed the localization of MIG-14::GFP and hTAC::GFP after knock-down of the individual candidates. We obtained RNAi bacterial strains of *arl-1*, *gbf-1*, *mon-2* and *unc-11*. We found that *gbf-1(RNAi)* caused an accumulation of hTAC::GFP in the cytoplasm (S15 Fig) and about 60% of the *arl-1* RNAi treated worms showed a large decrease in the expression level of hTAC::GFP. The basolateral membrane localization of hTAC::GFP were not affected by any of them (S15 Fig). *mon-2(RNAi)* abolished specifically the lateral membrane localization of MIG-14::GFP along the circumferential (S16 Fig; vertical lines). RNAi of the other three genes had no obvious effect on MIG-14::GFP (S16 Fig). Together, these results neither prove nor reject the possibility of *sgk-1* regulating membrane trafficking through these four candidates.

### GBF-1 function is required for proper SGK-1 localization

**Fig 10:**
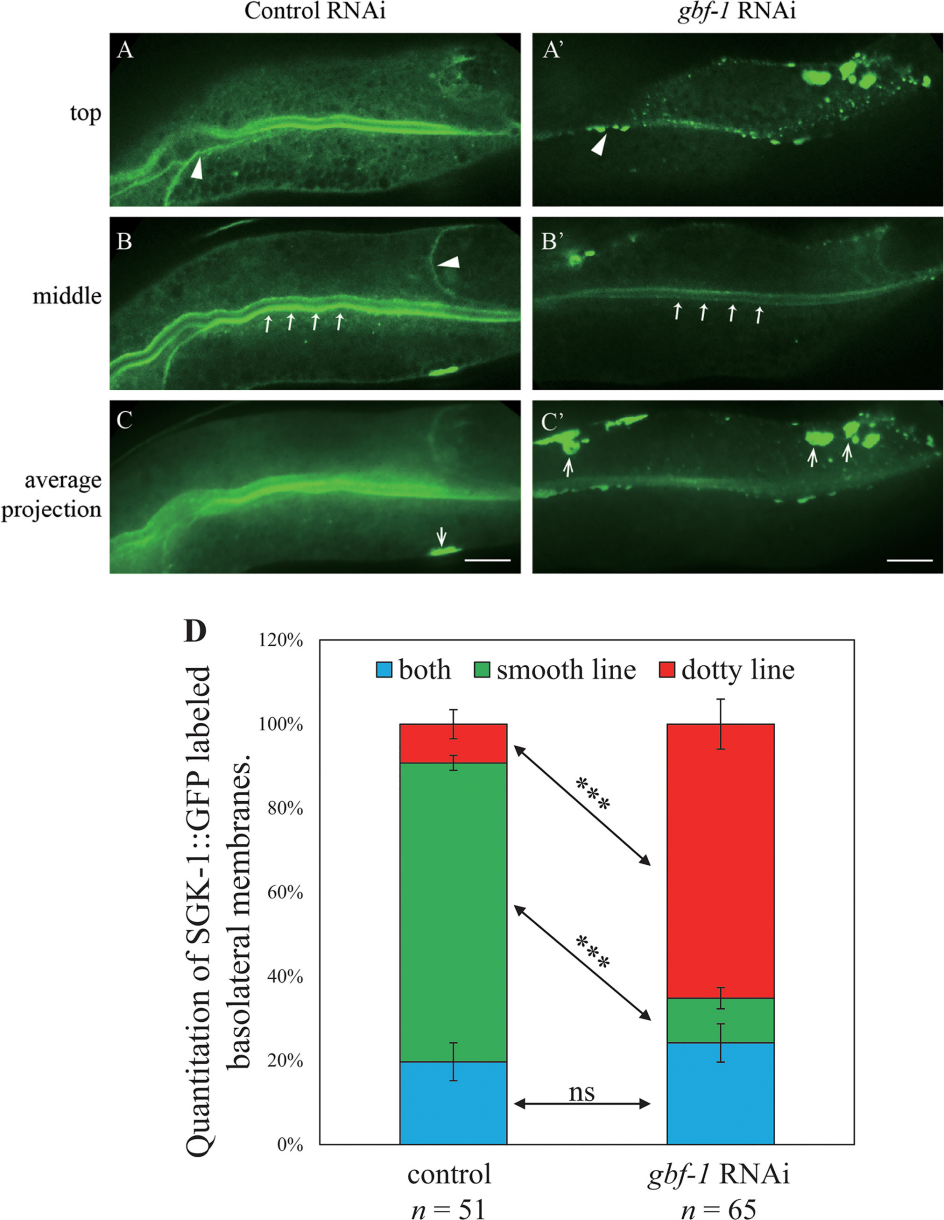
SGK-1::GFP lining the basolateral membrane of intestinal cells was affected by *gbf-1* RNAi. Confocal images of intestinal cells of worms expressing SGK-1::GFP treated with control RNAi (A, B, C) or *gbf-1* RNAi (A’, B’, C’). Scale bar represents 10 μm. (D) Quantitation of SGK-1::GFP labeled basolateral membranes. Some worms show dotted basolateral membranes in the top plane and smooth lateral membranes in the middle plane and they are named as “both”. *** *P* value <0.001, ns: non-significant (Student’s *t*-test).

Among the five candidate proteins, the ArfGEF protein GBF-1 has been characterized in intracellular trafficking in *C*. *elegans* [50]. The largest pool of GBF-1 resides at the cis-Golgi, and GBF-1 is required for endosome-mediated membrane traffic and the normal morphology of the Golgi and ER [50]. Thus, like SGK-1, GBF-1 acts at different stages in trafficking pathways. Therefore, we checked whether GBF-1 and SGK-1 would affect each other. *sgk-1(RNAi)* had no effect on steady-state GBF-1 localization (data not shown). In contrast, *gbf-1(RNAi)* affected the localization of SGK-1. SGK-1::GFP displayed a smooth, continuous distribution along the cortex of basolateral membrane of the intestinal cells. Upon *gbf-1* RNAi, SGK-1::GFP at the cortex of basolateral membrane changed to small punctate structures (Fig 10). Likewise the apical localization of SGK-1::GFP was affected by *gbf-1* RNAi, since the staining was strongly reduced in the RNAi-treated animals (Fig 10, panels B and B’, pointed arrows). Meanwhile, there were more bright, large-sized SGK-1::GFP aggregates on the basolateral side of the intestinal cells (Fig 10C’, open arrows). Such aggregates were observed in control animals only occasionally (Fig 10C, open arrows). The overall fluorescence intensity of SGK-1::GFP appeared not affected by *gbf-1* RNAi (S17 Fig), probably because the increase of SGK-1::GFP aggregates offset the decrease in the plasma membrane associated SGK-1::GFP. Considering that GBF-1 co-immunoprecipitated with SGK-1 (Table 1), this result suggests that GBF-1 may be required for SGK-1 function near the basolateral membrane by either being involved in the exocytosis of a SGK-1 binding protein at the plasma membrane or through a role in lipid homeostasis. Alternatively, SGK-1 may directly require GBF-1 GEF function.

## Discussion

In this study, we have found that SGK-1 associates *in vivo* with five proteins functioning in membrane trafficking. In *C*. *elegans*, both COGC-3 and GBF-1 reside on the Golgi, and the same can be said for ARL-1 and MON-2 in other species [48, 50, 52, 54]. The N-terminal region of Mon2p, which is conserved from yeast to human, is sufficient to bind the Golgi membrane where Mon2p acts as a scaffold protein [52]. Thus, we speculate that SGK-1 may be recruited onto the Golgi membrane to regulate membrane trafficking, although no apparent co-localization was detected between SGK-1::GFP and the Golgi marker mCHERRY::MANS. Further studies are needed to determine whether SGK-1 is recruited to the Golgi membrane under certain conditions.

The ArfGEF protein GBF-1 is localized in close proximity to the Golgi and the ER-exit sites, and is required for the normal morphology of ER [50]. In this study, we found that GBF-1 co-immunoprecipitated with SGK-1 (Table 1) and the loss of *sgk-1* induced unfolded protein response in ER (Fig 8). Therefore, although SGK-1::GFP is seen diffusely distributed in the cytoplasm for the most part (S13 Fig), we speculate that SGK-1 may be transiently recruited onto intracellular membranes by GBF-1 and functions with or through GBF-1 to regulate trafficking of cargo proteins in or out of ER. Supporting this idea, the smooth distribution of SGK-1::GFP along the basolateral membrane was disrupted by *gbf-1* RNAi (Fig 10). It is conceivable that SGK-1 may phosphorylate GBF-1 and alter its activity at the periphery of ER. More studies are needed to characterize the interaction between SGK-1 and GBF-1 and dissect the mechanism by which these two proteins regulate protein trafficking around ER.

Transcytosis allows for the exchange of cargoes between the apical and basolateral membranes and it is critical for the establishment and maintenance of the cell polarity [55]. Although there is no obvious membrane-targeting signal on SGK-1, this protein is seen near or on the plasma membrane in intestinal cells (S13 Fig). The concentration of SGK-1::GFP is higher in the cortex of apical membrane than that of basolateral membrane. Curiously, deletion of *sgk-1* affected basolateral recycling more severely than apical recycling, suggesting that SGK-1 activity is more important at basolateral membrane than apical membrane where there is more SGK-1 under normal situations. Also, *gbf-1* RNAi disrupted the smooth appearance of SGK-1::GFP lining the basolateral membrane, but not that lining the apical membrane (Fig 10). It would be interesting to determine whether SGK-1 and GBF-1 are both involved in transcytosis to coordinate directed movement of cargoes.

Ypk1—the yeast homologue of SGK-1—activates serine palmitoyl-CoA acyltransferase (SPT), which catalyzes the first and the key step of *de novo* synthesis of ceramide [25, 26]. Recently, Ypk1 is also reported to activate ceramide synthase [26]. We suspect that similar to yeast Ypk1, *C*. *elegans* SGK-1 may play a positive role in ceramide biosynthesis. In *C*. *elegans*, two types of complex sphingolipids, sphingomyelins and glycosphingolipids, are derived from ceramide and both contain an unusual, branched-chain sphingonoid base known as d17iso [56]. It is suggested that a particular species of sphingolipid called d17iso-GluCer or its derivative activates the TORC1 complex to promote larval development [57]. Inactivating the enzymes along the biosynthesis pathway of d17iso-GluCer arrests *C*. *elegans* at the L1 stage [57] or causes an uncharacterized lethal phenotype [58]. Our observation that *sgk-1(null)* exacerbated the developmental phenotype of *cgt-1/cgt-3* RNAi (Fig 6) is consistent with SGK-1 playing a positive role in ceramide synthesis, because this phenotype can be explained by a reduction of d17iso-GluCer by *cgt-1/cgt-3* RNAi, which is moderate in the wild-type background but greatly worsened by a reduction of ceramide, the precursor of d17iso-GluCer, in *sgk-1* null animals. Also, we speculate that *cgt-1/cgt-3* RNAi alleviated the trafficking phenotype of *sgk-1(null)* animals (Figs 4 and 5) by diverting the limited supply of ceramide to synthesizing sphingomyelins. In *sgk-1* mutants, sphingomyelins may be reduced the most among all complex sphingolipids, which leads to impaired membrane trafficking. Alternatively, all complex sphingolipids may be reduced to similar levels, but the trafficking phenotype may be more sensitive to a reduction in sphingomyelins than in glycosphingolipids. Following the hypothesis that SGK-1 regulates membrane trafficking by regulating ceramide synthesis, we expected that Myriocin, an inhibitor of SPT, would alter the membrane localization of MIG-14::GFP and hTAC::GFP, but it did not (S11 and S12 Figs). Possibly, Myriocin was not delivered efficiently enough to show an effect due to the tight barrier of the worm cuticle. It remains to be tested whether inactivating *sptl-1*, *sptl-2* and *sptl-3*, the SPT genes in *C*. *elegans*, would cause the same trafficking phenotype observed in *sgk-1* mutants.

## Supporting Information

S1 Fig Autofluorescence of wild type and *sgk-1(ok538)* mutant animals in red, green and blue channels. Confocal images of intestinal cells of wild type worms (A, A’, A”, A”‘) and *sgk-1(ok538)* mutant animals (B, B’, B”, B”‘) in red channel (A’ B’), green channel (A”, B”) and blue channel (A”‘, B”‘). Scale bars: 5 μm.
(EPS)

S2 Fig Autofluorescence of wild type and *sgk-1(ok538)* mutant animals in red, green and blue channels with different imaging conditions. Confocal images of intestinal cells of wild type worms (A to I) and *sgk-1(ok538)* mutant animals (A’ to I’) in red channel (A to C, A’ to C’), green channel (D to F, D’ to F’) and blue channel (G to I, G’ to I’) in “low” condition (A, D, G, A’, D’, G’), “middle” condition (B, E, H, B’, E’, H’) and “high” condition (C, F, I, C’, F’, I’). Low, middle, high denote three different settings of pinhole size and detector gain. High means larger number and low means smaller number. Scale bars: 5 μm.
(EPS)

S3 Fig Colocalization of MIG-14::GFP and autofluorescence in wild type worms. Confocal images of intestinal cells of wild type worms in green channel showing MIG-14::GFP (A, B) and blue channel (A’, B’) indicating the autofluorescence. The basolateral and apical membranes are indicated by arrows and arrowheads, respectively. The autofluorescent spots were different in shape and size from the MIG-14::GFP positive structures and the GFP signals in Fig 1 did not colocalize with the autofluorescence in blue channel. Scale bars: 5 μm.
(EPS)

S4 Fig Colocalization of MIG-14::GFP and autofluorescence in *sgk-1* null mutant. Confocal images of intestinal cells of *sgk-1(ok538)* mutant worms in green channel showing MIG-14::GFP (A, B) and blue channel (A’, B’) indicating the autofluorescence. The autofluorescent spots were different in shape and size from the MIG-14::GFP positive structures and the GFP signals in Fig 1 did not colocalize with the autofluorescence in blue channel. Scale bars: 5 μm.
(EPS)

S5 Fig Colocalization of MIG-14::GFP and autofluorescence in *sgk-1* null mutant. Confocal images of intestinal cells of *sgk-1(mg455)* mutant worms in green channel showing MIG-14::GFP (A, B) and blue channel (A’, B’) indicating the autofluorescence. The autofluorescent spots were different in shape and size from the MIG-14::GFP positive structures and the GFP signals in Fig 1 did not colocalize with the autofluorescence in blue channel. Scale bars: 5 μm.
(EPS)

S6 Fig Colocalization of hTAC::GFP and autofluorescence in wild type worms. Confocal images of intestinal cells of wild type worms in green channel showing hTAC::GFP (A, B) and blue channel (A’, B’) indicating the autofluorescence. The autofluorescent spots were different in shape and size from the hTAC::GFP positive structures and the GFP signals in Fig 3 did not colocalize with the autofluorescence in blue channel. Scale bars: 5 μm.
(EPS)

S7 Fig Colocalization of hTAC::GFP and autofluorescence in *sgk-1* null mutant. Confocal images of intestinal cells of *sgk-1(ok538)* mutant worms in green channel showing hTAC::GFP (A, B) and blue channel (A’, B’) indicating the autofluorescence. The autofluorescent spots were different in shape and size from the hTAC::GFP positive structures and the GFP signals in Fig 3 did not colocalize with the autofluorescence in blue channel. Scale bars: 5 μm.
(EPS)

S8 Fig Colocalization of hTAC::GFP and autofluorescence in *sgk-1* null mutant. Confocal images of intestinal cells of *sgk-1(mg455)* mutant worms in green channel showing hTAC::GFP (A, B) and blue channel (A’, B’) indicating the autofluorescence. The autofluorescent spots were different in shape and size from the hTAC::GFP positive structures and the GFP signals in Fig 3 did not colocalize with the autofluorescence in blue channel. Scale bars: 5 μm.
(EPS)

S9 Fig Colocalization of hTAC::GFP and RFP::RAB-5 in wild type and *sgk-1* null mutants. Confocal images of the wild type (A, A’, A”) and *sgk-1(null)* (B, B’, B”) intestinal cells expressing hTAC::GFP (A, B) or RFP::RAB-5 (A’, B’). Insets show magnified areas (× 2.5). The overlapped and non-overlapped regions are indicated by arrows and arrowheads, respectively. Scale bars: 5 μm.
(EPS)

S10 Fig The expression pattern of GLUT1::GFP in young adult worms. Confocal images of intestinal cells of young adult wild type (A) and *sgk-1(ok538)* (B) worms expressing GLUT1::GFP. The basolateral membranes are indicated by arrows. Scale bars: 5 μm.
(EPS)

S11 Fig The localization patterns of hTAC::GFP are unaffected by Myriocin. Confocal images of wild-type intestinal cells treated with control (A, A’), 4.2 μM of myriocin (B, B’), 25.2 μM of myriocin (C, C’) and 50.4 μM of myriocin (D, D’) expressing hTAC::GFP. The basolateral membranes were indicated by arrows. Scale bars: 5 μm.
(EPS)

S12 Fig The localization patterns of MIG-14::GFP are unaffected by Myriocin. Confocal images of wild-type intestinal cells treated with control (A, A’), 4.2 μM of myriocin (B, B’), 25.2 μM of myriocin (C, C’) and 50.4 μM of myriocin (D, D’) expressing MIG-14::GFP. The basolateral membranes were indicated by arrows. Scale bars: 5 μm.
(EPS)

S13 Fig Expression pattern of SGK-1::GFP. SGK-1::GFP expression is seen in embryos, all larval stages and throughout adulthood. SGK-1::GFP is highly expressed in the intestine and the head and tail neurons, as described previously [28]. The apical and basolateral membranes were indicated by arrow heads and arrows. Scale bars: 10 μm.
(EPS)

S14 Fig Yeast two-hybrid assay of SGK-1 and candidate binding proteins. Yeast cells were co-transformed with a GAL4BD-bait plasmid and a GAL4AD-prey plasmid as indicated. Among the double transformants (grown in the absence of Leu and Trp, or–LW) only two (AD-SGK-1/BD-COGC-3 and AD-SGK-1/BD-MON-2N) displayed positive interactions as they grew in the absence of Leu, Trp and His (–LWH). The apparent positive interaction of AD-SGK-1/BD-UNC-11 was false because BD-UNC-11 showed self-activating activity independent of the prey (AD-SGK-1 or AD-Xrc4). Xrc4 is a negative control; it is a nonhomologous end joining factor from *S*. *pombe* [59] and is not expected to interact with *C*. *elegans* proteins. MON-2N: 1–800 aa; MON-2C: 801–1648 aa; GBF-1N: 1–1147 aa; GBF-1C: 1148–1975 aa.
(EPS)

S15 Fig Localization of hTAC::GFP upon RNAi of candidate interacting proteins of SGK-1. Confocal images of intestinal cells of wild type worms treated with control RNAi (A, A’), *arl-1* RNAi (B, B’), *gbf-1* RNAi (C, C’), *mon-2* RNAi (D, D’), and *unc-11* RNAi (E, E’) and *sgk-1(ok538)* mutants (F, F’) expressing hTAC::GFP. Scale bars: 5 μm.
(EPS)

S16 Fig Localization of MIG-14::GFP upon RNAi of candidate interacting proteins of SGK-1. Confocal images of intestinal cells of wild type worms treated with control RNAi (A, A’), *arl-1* RNAi (B, B’), *gbf-1* RNAi (C, C’), *mon-2* RNAi (D, D’), and *unc-11* RNAi (E, E’) and *sgk-1(ok538)* mutants (F, F’) expressing MIG-14::GFP. The basolateral membranes are indicated by arrows. Scale bars: 5 μm.
(EPS)

S17 Fig Overall fluorescence intensity of SGK-1::GFP with *gbf-1* RNAi. The overall fluorescence intensity of SGK-1::GFP was unaffected by *gbf-1* RNAi treatment. * *P* value <0.05 (Student’s *t*-test).
(EPS)

S1 File Worms expressing SGK-1::GFP treated with control RNAi showing smooth basolateral membrane. (AVI)

S2 File Worms expressing SGK-1::GFP treated with *gbf-1* RNAi showing dotty basolateral membrane. (AVI)

S3 File Worms expressing SGK-1::GFP treated with gbf-1 RNAi showing “both” basolateral membrane. (AVI)

S1 Table Summary of pinhole size, detector gain, offset, and laser intensity settings for each figure. (XLSX)

## References

[1] Firestone GL, Giampaolo JR, O’Keeffe BA. Stimulus-dependent regulation of serum and glucocorticoid inducible protein kinase (SGK) transcription, subcellular localization and enzymatic activity. Cellular Physiology and Biochemistry. 2003;13(1):1–12. 12649597 10.1159/000070244

[2] Webster M, Goya L, Firestone G. Immediate-early transcriptional regulation and rapid mRNA turnover of a putative serine/threonine protein kinase. Journal of Biological Chemistry. 1993;268(16):11482–5. 8505283

[3] Webster MK, Goya L, Ge Y, Maiyar A, Firestone G. Characterization of sgk, a novel member of the serine/threonine protein kinase gene family which is transcriptionally induced by glucocorticoids and serum. Molecular and cellular biology. 1993;13(4):2031–40. 8455596 10.1128/mcb.13.4.2031-2040.1993 PMC359524

[4] Lang F, Artunc F, Vallon V. The physiological impact of the serum-and glucocorticoid-inducible kinase SGK1. Current opinion in nephrology and hypertension. 2009;18(5):439 10.1097/MNH.0b013e32832f125e 19584721 PMC2883450

[5] Lang F, Böhmer C, Palmada M, Seebohm G, Strutz-Seebohm N, Vallon V. (Patho) physiological significance of the serum-and glucocorticoid-inducible kinase isoforms. Physiological Reviews. 2006;86(4):1151–78. 17015487 10.1152/physrev.00050.2005

[6] Faresse N, Lagnaz D, Debonneville A, Ismailji A, Maillard M, Fejes-Toth G, et al Inducible kidney-specific Sgk1 knockout mice show a salt-losing phenotype. American Journal of Physiology-Renal Physiology. 2012;302(8):F977–F85. 10.1152/ajprenal.00535.2011 22301619

[7] Leong ML, Maiyar AC, Kim B, O'Keeffe BA, Firestone GL. Expression of the serum-and glucocorticoid-inducible protein kinase, Sgk, is a cell survival response to multiple types of environmental stress stimuli in mammary epithelial cells. Journal of Biological Chemistry. 2003;278(8):5871–82. 12488318 10.1074/jbc.M211649200

[8] Lu M, Wang J, Jones KT, Ives HE, Feldman ME, Yao L-j, et al mTOR complex-2 activates ENaC by phosphorylating SGK1. Journal of the American Society of Nephrology. 2010;21(5):811–8. 10.1681/ASN.2009111168 20338997 PMC2865740

[9] Brunet A, Park J, Tran H, Hu LS, Hemmings BA, Greenberg ME. Protein kinase SGK mediates survival signals by phosphorylating the forkhead transcription factor FKHRL1 (FOXO3a). Molecular and cellular biology. 2001;21(3):952–65. 11154281 10.1128/MCB.21.3.952-965.2001 PMC86685

[10] BelAiba RS, Djordjevic T, Bonello S, Artunc F, Lang F, Hess J, et al The Serum-and Glucocorticoid-Inducible Kinase Sgk-1 Is Involved in Pulmonary Vascular Remodeling Role in Redox-Sensitive Regulation of Tissue Factor by Thrombin. Circulation research. 2006;98(6):828–36. 16484615 10.1161/01.RES.0000210539.54861.27

[11] Vallon V, Wyatt AW, Klingel K, Huang DY, Hussain A, Berchtold S, et al SGK1-dependent cardiac CTGF formation and fibrosis following DOCA treatment. Journal of molecular medicine. 2006;84(5):396–404. 16604333 10.1007/s00109-005-0027-z

[12] Tai DJ, Su C-C, Ma Y-L, Lee EH. SGK1 phosphorylation of IκB kinase α and p300 up-regulates NF-κB activity and increases N-methyl-d-aspartate receptor NR2A and NR2B expression. Journal of Biological Chemistry. 2009;284(7):4073–89. 10.1074/jbc.M805055200 19088076

[13] Lang F, Vallon V. Serum-and glucocorticoid-inducible kinase 1 in the regulation of renal and extrarenal potassium transport. Clinical and experimental nephrology. 2012;16(1):73–80. 10.1007/s10157-011-0488-z 22038256

[14] Lang F, Shumilina E. Regulation of ion channels by the serum-and glucocorticoid-inducible kinase SGK1. The FASEB Journal. 2013;27(1):3–12. 23012321 10.1096/fj.12-218230

[15] Lang F, Stournaras C. Serum and glucocorticoid inducible kinase, metabolic syndrome, inflammation, and tumor growth. Hormones (Athens). 2013;12:160–71. 23933686 10.14310/horm.2002.1401

[16] Bomberger JM, Coutermarsh BA, Barnaby RL, Sato JD, Chapline MC, Stanton BA. Serum and Glucocorticoid-Inducible Kinase1 Increases Plasma Membrane wt-CFTR in Human Airway Epithelial Cells by Inhibiting Its Endocytic Retrieval. PloS one. 2014;9(2):e89599 10.1371/journal.pone.0089599 24586903 PMC3931797

[17] Guggino WB, Stanton BA. New insights into cystic fibrosis: molecular switches that regulate CFTR. Nature Reviews Molecular Cell Biology. 2006;7(6):426–36. 16723978 10.1038/nrm1949

[18] Goldstein JL, Anderson RG, Brown MS. Coated pits, coated vesicles, and receptor-mediated endocytosis. Nature. 1979;279(5715):679–85. 221835 10.1038/279679a0

[19] Maxfield FR, McGraw TE. Endocytic recycling. Nature Reviews Molecular Cell Biology. 2004;5(2):121–32. 15040445 10.1038/nrm1315

[20] Harford J, Bridges K, Ashwell G, Klausner RD. Intracellular dissociation of receptor-bound asialoglycoproteins in cultured hepatocytes. A pH-mediated nonlysosomal event. Journal of Biological Chemistry. 1983;258(5):3191–7. 6298227

[21] Mukherjee S, Ghosh RN, Maxfield FR. Endocytosis1997 1997-07-01 00:00:00. 759–803 p.

[22] Mellman I. Endocytosis and molecular sorting. Annual review of cell and developmental biology. 1996;12(1):575–625. 10.1146/annurev.cellbio.12.1.575 8970738

[23] Grant BD, Donaldson JG. Pathways and mechanisms of endocytic recycling. Nature Reviews Molecular Cell Biology. 2009;10(9):597–608. 10.1038/nrm2755 19696797 PMC3038567

[24] Bonifacino JS, Rojas R. Retrograde transport from endosomes to the trans-Golgi network. Nature Reviews Molecular Cell Biology. 2006;7(8):568–79. 16936697 10.1038/nrm1985

[25] Roelants FM, Breslow DK, Muir A, Weissman JS, Thorner J. Protein kinase Ypk1 phosphorylates regulatory proteins Orm1 and Orm2 to control sphingolipid homeostasis in Saccharomyces cerevisiae. Proceedings of the National Academy of Sciences. 2011;108(48):19222–7. 10.1073/pnas.1116948108 22080611 PMC3228448

[26] Muir A, Ramachandran S, Roelants FM, Timmons G, Thorner J. TORC2-dependent protein kinase Ypk1 phosphorylates ceramide synthase to stimulate synthesis of complex sphingolipids. Elife. 2014;3:e03779. 10.7554/eLife.03779 PMC4217029 25279700

[27] Futerman AH, Stieger B, Hubbard A, Pagano RE. Sphingomyelin synthesis in rat liver occurs predominantly at the cis and medial cisternae of the Golgi apparatus. Journal of Biological Chemistry. 1990;265(15):8650–7. 2187869

[28] Zhang H, Abraham N, Khan LA, Hall DH, Fleming JT, Göbel V. Apicobasal domain identities of expanding tubular membranes depend on glycosphingolipid biosynthesis. Nature Cell Biology. 2011;13(10):1189–201. 10.1038/ncb2328 21926990 PMC3249144

[29] Hertweck M, Göbel C, Baumeister R. < i> C. elegans SGK-1 Is the Critical Component in the Akt/PKB Kinase Complex to Control Stress Response and Life Span. Developmental cell. 2004;6(4):577–88. 15068796 10.1016/s1534-5807(04)00095-4

[30] Chen ATY, Guo C, Dumas KJ, Ashrafi K, Hu PJ. Effects of Caenorhabditis elegans sgk‐1 mutations on lifespan, stress resistance, and DAF‐16/FoxO regulation. Aging cell. 2013;12(5):932–40. 10.1111/acel.12120 23786484 PMC3824081

[31] Chen D, Li PW-L, Goldstein BA, Cai W, Thomas EL, Chen F, et al Germline Signaling Mediates the Synergistically Prolonged Longevity Produced by Double Mutations in< i> daf-2 and< i> rsks-1 in< i> C. elegans. Cell reports. 2013;5(6):1600–10. 10.1016/j.celrep.2013.11.018 24332851 PMC3904953

[32] Xiao R, Zhang B, Dong Y, Gong J, Xu T, Liu J, et al A Genetic Program Promotes< i> C. elegans Longevity at Cold Temperatures via a Thermosensitive TRP Channel. Cell. 2013;152(4):806–17. 10.1016/j.cell.2013.01.020 23415228 PMC3594097

[33] Li X, Chen B, Yoshina S, Cai T, Yang F, Mitani S, et al Inactivation of Caenorhabditis elegans aminopeptidase DNPP-1 restores endocytic sorting and recycling in tat-1 mutants. Molecular biology of the cell. 2013;24(8):1163–75. 10.1091/mbc.E12-10-0730 23427264 PMC3623637

[34] Kamath RS, Fraser AG, Dong Y, Poulin G, Durbin R, Gotta M, et al Systematic functional analysis of the Caenorhabditis elegans genome using RNAi. Nature. 2003;421(6920):231–7. 12529635 10.1038/nature01278

[35] Xu T, Venable J, Park SK, Cociorva D, Lu B, Liao L, et al., editors. ProLuCID, a fast and sensitive tandem mass spectra-based protein identification program. Molecular & Cellular Proteomics; 2006: AMER SOC BIOCHEMISTRY MOLECULAR BIOLOGY INC 9650 ROCKVILLE PIKE, BETHESDA, MD 20814–3996 USA.

[36] Tabb DL, McDonald WH, Yates JR. DTASelect and Contrast: tools for assembling and comparing protein identifications from shotgun proteomics. Journal of proteome research. 2002;1(1):21–6. 12643522 10.1021/pr015504q PMC2811961

[37] Sowa ME, Bennett EJ, Gygi SP, Harper JW. Defining the human deubiquitinating enzyme interaction landscape. Cell. 2009;138(2):389–403. 10.1016/j.cell.2009.04.042 19615732 PMC2716422

[38] Vizcaíno JA, Deutsch EW, Wang R, Csordas A, Reisinger F, Rios D, et al ProteomeXchange provides globally coordinated proteomics data submission and dissemination. Nature biotechnology. 2014;32(3):223–6. 10.1038/nbt.2839 24727771 PMC3986813

[39] Takada R, Satomi Y, Kurata T, Ueno N, Norioka S, Kondoh H, et al Monounsaturated fatty acid modification of Wnt protein: its role in Wnt secretion. Developmental cell. 2006;11(6):791–801. 17141155 10.1016/j.devcel.2006.10.003

[40] Yang P-T, Lorenowicz MJ, Silhankova M, Coudreuse DY, Betist MC, Korswagen HC. Wnt signaling requires retromer-dependent recycling of MIG-14/Wntless in Wnt-producing cells. Developmental cell. 2008;14(1):140–7. 18160347 10.1016/j.devcel.2007.12.004

[41] Pan C-L, Baum PD, Gu M, Jorgensen EM, Clark SG, Garriga G. < i> C. elegans AP-2 and Retromer Control Wnt Signaling by Regulating MIG-14/Wntless. Developmental cell. 2008;14(1):132–9. 18160346 10.1016/j.devcel.2007.12.001 PMC2709403

[42] Shi A, Sun L, Banerjee R, Tobin M, Zhang Y, Grant BD. Regulation of endosomal clathrin and retromer‐mediated endosome to Golgi retrograde transport by the J‐domain protein RME‐8. The EMBO journal. 2009;28(21):3290–302. 10.1038/emboj.2009.272 19763082 PMC2776105

[43] Chen B, Jiang Y, Zeng S, Yan J, Li X, Zhang Y, et al Endocytic sorting and recycling require membrane phosphatidylserine asymmetry maintained by TAT-1/CHAT-1. PLoS genetics. 2010;6(12):e1001235 10.1371/journal.pgen.1001235 21170358 PMC3000356

[44] Chen CC-H, Schweinsberg PJ, Vashist S, Mareiniss DP, Lambie EJ, Grant BD. RAB-10 is required for endocytic recycling in the Caenorhabditis elegans intestine. Molecular biology of the cell. 2006;17(3):1286–97. 16394106 10.1091/mbc.E05-08-0787 PMC1382317

[45] Eyster CA, Higginson JD, Huebner R, Porat‐Shliom N, Weigert R, Wu WW, et al Discovery of New Cargo Proteins that Enter Cells through Clathrin‐Independent Endocytosis. Traffic. 2009;10(5):590–9. 10.1111/j.1600-0854.2009.00894.x 19302270 PMC2854272

[46] Nomura KH, Murata D, Hayashi Y, Dejima K, Mizuguchi S, Kage-Nakadai E, et al Ceramide glucosyltransferase of the nematode Caenorhabditis elegans is involved in oocyte formation and in early embryonic cell division. Glycobiology. 2011;21(6):834–48. 10.1093/glycob/cwr019 21325339

[47] Rothbauer U, Zolghadr K, Muyldermans S, Schepers A, Cardoso MC, Leonhardt H. A versatile nanotrap for biochemical and functional studies with fluorescent fusion proteins. Molecular & Cellular Proteomics. 2008;7(2):282–9. 17951627 10.1074/mcp.M700342-MCP200

[48] Haynes LP, Sherwood MW, Dolman NJ, Burgoyne RD. Specificity, Promiscuity and Localization of ARF Protein Interactions with NCS‐1 and Phosphatidylinositol‐4 Kinase‐IIIβ. Traffic. 2007;8(8):1080–92. 17555535 10.1111/j.1600-0854.2007.00594.x PMC2492389

[49] Morinaga N, Tsai S-C, Moss J, Vaughan M. Isolation of a brefeldin A-inhibited guanine nucleotide-exchange protein for ADP ribosylation factor (ARF) 1 and ARF3 that contains a Sec7-like domain. Proceedings of the National Academy of Sciences. 1996;93(23):12856–60. 8917509 10.1073/pnas.93.23.12856 PMC24010

[50] Ackema KB, Sauder U, Solinger JA, Spang A. The ArfGEF GBF-1 is required for ER structure, secretion and endocytic transport in C. elegans. PloS one. 2013;8(6):e67076 23840591 10.1371/journal.pone.0067076 PMC3686754

[51] Suvorova ES, Kurten RC, Lupashin VV. Identification of a human orthologue of Sec34p as a component of the cis-Golgi vesicle tethering machinery. Journal of Biological Chemistry. 2001;276(25):22810–8. 11292827 10.1074/jbc.M011624200

[52] Efe JA, Plattner F, Hulo N, Kressler D, Emr SD, Deloche O. Yeast Mon2p is a highly conserved protein that functions in the cytoplasm-to-vacuole transport pathway and is required for Golgi homeostasis. Journal of cell science. 2005;118(20):4751–64. 16219684 10.1242/jcs.02599

[53] Nonet ML, Holgado AM, Brewer F, Serpe CJ, Norbeck BA, Holleran J, et al UNC-11, a Caenorhabditis elegans AP180 homologue, regulates the size and protein composition of synaptic vesicles. Molecular biology of the cell. 1999;10(7):2343–60. 10397769 10.1091/mbc.10.7.2343 PMC25452

[54] Kubota Y, Sano M, Goda S, Suzuki N, Nishiwaki K. The conserved oligomeric Golgi complex acts in organ morphogenesis via glycosylation of an ADAM protease in C. elegans. Development. 2006;133(2):263–73. 16354716 10.1242/dev.02195

[55] Apodaca G, Katz LA, Mostov KE. Receptor-mediated transcytosis of IgA in MDCK cells is via apical recycling endosomes. The Journal of cell biology. 1994;125(1):67–86. 8138576 10.1083/jcb.125.1.67 PMC2120019

[56] Chitwood DJ, Lusby WR, Thompson MJ, Kochansky JP, Howarth OW. The glycosylceramides of the nematodeCaenorhabditis elegans contain an unusual, branched-chain sphingoid base. Lipids. 1995;30(6):567–73. 7651085 10.1007/BF02537032

[57] Zhu H, Shen H, Sewell AK, Kniazeva M, Han M. A novel sphingolipid-TORC1 pathway critically promotes postembryonic development in Caenorhabditis elegans. Elife. 2013;2:e00429 10.7554/eLife.00429 23705068 PMC3660743

[58] Menuz V, Howell KS, Gentina S, Epstein S, Riezman I, Fornallaz-Mulhauser M, et al Protection of C. elegans from anoxia by HYL-2 ceramide synthase. Science. 2009;324(5925):381–4. 10.1126/science.1168532 19372430

[59] Li J, Yu Y, Suo F, Sun L-L, Zhao D, Du L-L. Genome-wide Screens for Sensitivity to Ionizing Radiation Identify the Fission Yeast Nonhomologous End Joining Factor Xrc4. G3: Genes| Genomes| Genetics. 2014;4(7):1297–306. 10.1534/g3.114.011841 24847916 PMC4455778

