# Correction: Serum- and Glucocorticoid-Inducible Kinase-1 (SGK-1) Plays a Role in Membrane Trafficking in Caenorhabditis elegans

**DOI:** 10.1371/journal.pone.0163398

**Published:** 2016-09-15

**Authors:** Ming Zhu, Gang Wu, Yu-Xin Li, Julia Kathrin Stevens, Chao-Xuan Fan, Anne Spang, Meng-Qiu Dong

Fig 6B appears incorrectly as a duplicate of Fig 6C in the published article. Please see the correct Fig 6 and its legend here.

**Fig 6:**
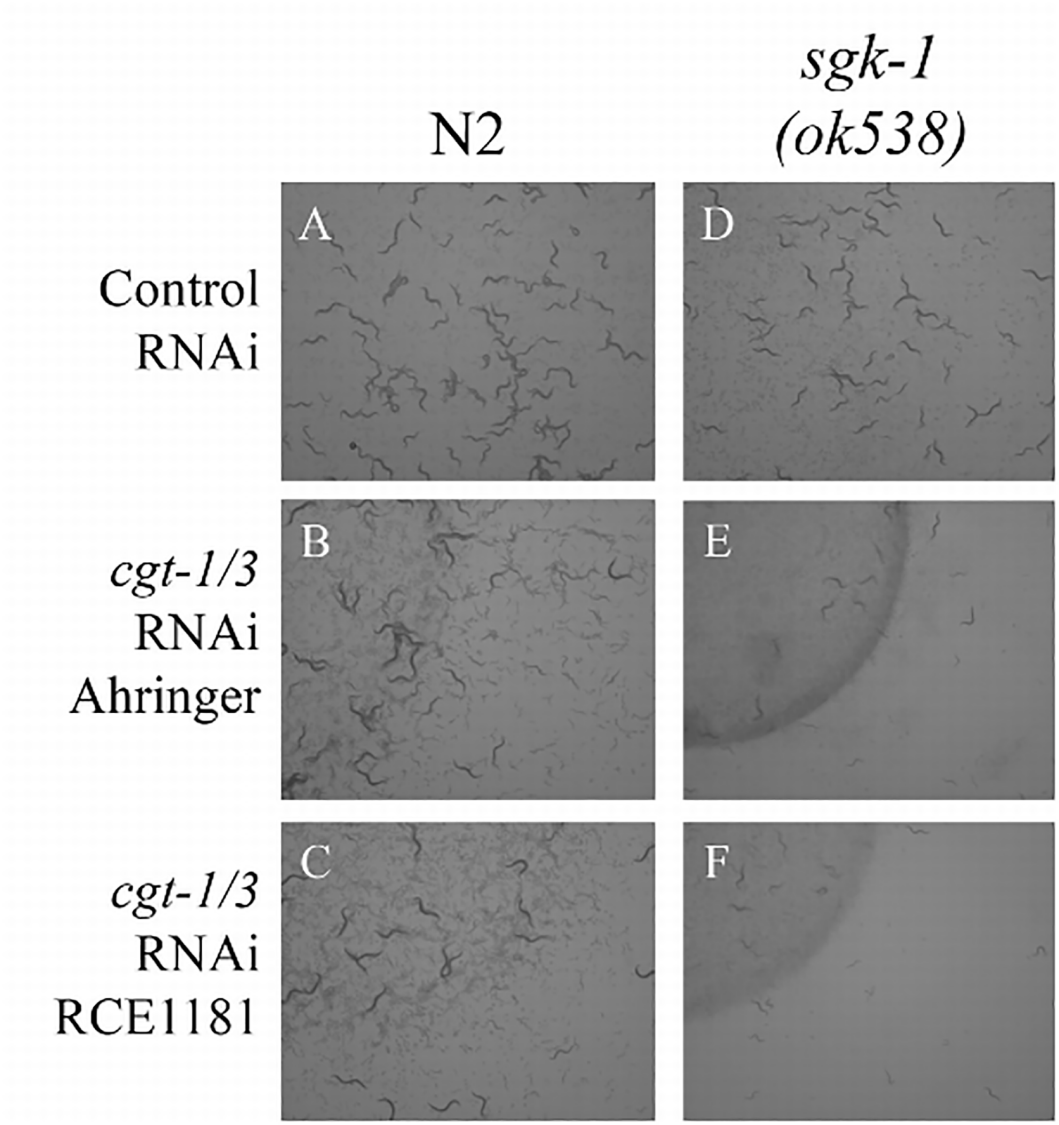
*sgk-1* mutant worms treated with *cgt-1/3* double RNAi developed very slowly. Images of wild type (A, B, C) and *sgk-1(ok538)* mutant animals (D, E, F) treated with control RNAi (A, D) and *cgt-1/3* double RNAi from Ahringer library (B, E) and RCE1181 library (C, F). The experiments started with 5 gravid adult worms on each plate and the plates were imaged 5 days later.
